# DNA damage burden causes selective CUX2 neuron loss in neuroinflammation

**DOI:** 10.1038/s41586-026-10310-3

**Published:** 2026-04-01

**Authors:** Laura Morcom, Wenlong Xia, Zhaoyang Xu, Yashika Awasthi, Celine Geywitz, Matthew O. Ellis, Tomas Noli, Amel Zulji, Daniel Yamamoto, Gemma C. Girdler, Li Kai, Keying Zhu, Mingming Wei, Xiao-Yan Tang, Kimberly K. Hoi, Julio Gonzalez-Maya, Greg J. Duncan, Adrien M. Vaquie, Diana Gold Diaz, Riki Kawaguchi, Erdong Liu, Yu Sun, Denny Yang, Gregory D. Jordan, I-Ling Lu, Staffan Holmqvist, Theresa Bartels, Katherine Ridley, Jennifer Ja-Yoon Choi, Santos J. Franco, Eric J. Huang, Ben Emery, Daniel Geschwind, Lucas Schirmer, Gabriel Balmus, Brian Popko, Stephen P. J. Fancy, David H. Rowitch

**Affiliations:** 1 Cambridge Stem Cell Institute, University of Cambridge, Cambridge, UK; 2 UK Dementia Research Institute at the University of Cambridge, University of Cambridge, Cambridge, UK; 3 Department of Paediatrics, University of Cambridge, Cambridge, UK; 4 Department of Clinical Neurosciences, University of Cambridge, Cambridge, UK; 5 Division of Neuroimmunology and Glial Biology, Department of Neurology, University of California San Francisco, San Francisco, CA USA; 6 Department of Pediatrics, Cedars-Sinai Guerin Children’s, Los Angeles, CA USA; 7 Department of Neurosurgery, Cedars-Sinai Guerin Children’s, Los Angeles, CA USA; 8 Department of Neurology, Feinberg School of Medicine, Northwestern University, Chicago, IL USA; 9 Division of Neuroimmunology, Department of Neurology, Medical Faculty Mannheim, Heidelberg University, Mannheim, Germany; 10 Department of Neurology, Jungers Center for Neurosciences Research, Oregon Health & Science University, Portland, OR USA; 11 Semel Institute for Neuroscience and Human Behavior, David Geffen School of Medicine, University of California Los Angeles, Los Angeles, CA USA; 12 Department of Neurology, University of California Los Angeles, Los Angeles, CA USA; 13 The Eli and Edythe Broad Center of Regeneration Medicine and Stem Cell Research, University of California San Francisco, San Francisco, CA USA; 14 Department of Pediatrics, Division of Neonatology, University of California San Francisco, San Francisco, CA USA; 15 Department of Pathology, University of California San Francisco, San Francisco, CA USA; 16 Department of Pediatrics, Section of Developmental Biology, University of Colorado—Anschutz Medical Campus, Denver, CO USA; 17 Jungers Center for Neurosciences Research, Department of Neurology, Oregon Health & Science University, Portland, OR USA; 18 Program in Neurogenetics, Departments of Neurology and Human Genetics, Institute of Precision Health, David Geffen School of Medicine, University of California Los Angeles, Los Angeles, CA USA; 19 Center for Autism Research and Treatment, Department of Psychiatry and Semel Institute, University of California Los Angeles, Los Angeles, CA USA; 20 Interdisciplinary Center for Neurosciences, Heidelberg University, Heidelberg, Germany; 21 Center for Translational Neuroscience and Institute for Innate Immunoscience, Medical Faculty Mannheim, Heidelberg University, Mannheim, Germany; 22 Department of Molecular Neuroscience, Transylvanian Institute of Neuroscience, Cluj-Napoca, Romania

**Keywords:** Multiple sclerosis, Experimental models of disease

## Abstract

Neurodegeneration shows regional and cell-type-specific patterns in ageing and disease^1^, but the underlying mechanisms for cell-type-specific neuronal losses remain poorly understood. Previous studies have shown that upper cortical layer thinning occurs in progressive human multiple sclerosis (MS) and that cortical layer 2 and layer 3 (L2/3) excitatory neurons (L2/3ENs) that express CUT-like homeobox 2 (*CUX2*) are selectively vulnerable to degeneration^2^. Here we report that L2/3ENs within MS cortical lesions have an elevated DNA damage burden. DNA damage and selective loss of L2/3ENs were recapitulated in diverse mouse models of demyelination and pan-cortical inflammation, confirming their intrinsic vulnerability. Functions of *Cux2* and activating transcription factor 4 (*Atf4*) were essential for resilience of L2/3ENs during postnatal neuroinflammation, acting in neurons to enhance DNA double-strand break repair. Interferon-γ, a cytokine implicated in MS pathogenesis^3,4^, was sufficient to elevate levels of reactive oxygen species, leading to DNA damage-mediated neuronal death in vitro, and caused selective depletion of L2/3 neurons in mice. These findings indicate that DNA damage burden and inadequate repair in CUX2^+^ L2/3ENs contributes to selective vulnerability in neuroinflammatory injury.

## Abstract (web summary)

DNA damage burden and inadequate repair in CUX2^+^ cortical layer 2/3 excitatory neurons contributes to selective vulnerability in neuroinflammatory injury.

## Main

Ageing and neurodegeneration are linked to the buildup of DNA damage in both glial cells and neurons^5–7^. Such damage can originate from internal sources like stress induced by metabolic by-products (for example, reactive oxygen species (ROS), advanced glycation end products and alkylation species) or normal cellular activities (such as transcription and replication)^5^. Moreover, DNA damage can arise from external factors such as cosmic ionizing radiation, diet, pollution or chemotherapy^5^. If left unrepaired, primary forms of DNA damage, including single-stranded breaks (SSBs), double-stranded breaks (DSBs) and oxidative base lesions, compromise neuronal function and lead to cell death. Neurons mount a DNA damage response (DDR), which involves repair pathways such as base-excision repair (BER) for SSBs and oxidative damage, transcription-coupled repair for DNA lesions blocking transcription, mismatch repair for correcting single base-pair mismatches or insertion–deletion loops, and error-prone non-homologous end joining (NHEJ) for DSBs that are critical for maintaining genomic stability^7^.

**Fig. 1:**
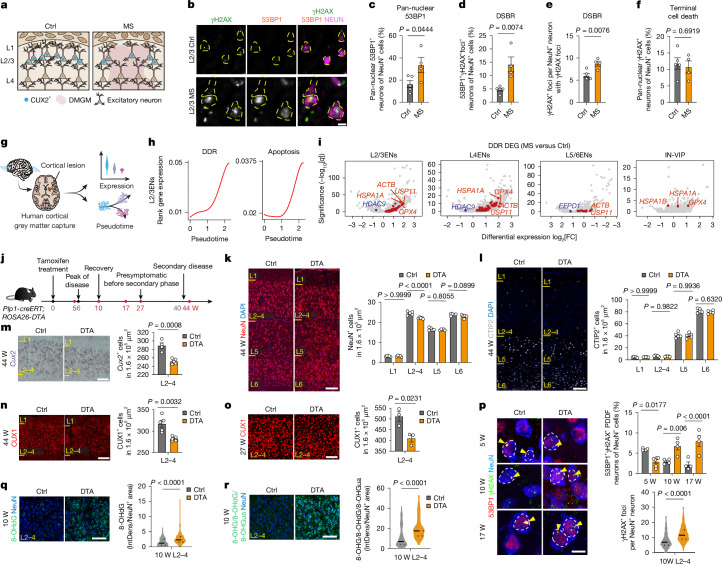
DNA damage and response in human MS and DTA mice. **a**, Schematic of cortical neuron layers in control (Ctrl) and demyelinated grey matter (DMGM) in MS. **b**–**f**, Immunohistochemistry (**b**) and quantification (**c**–**f**) of pan-nuclear 53BP1 (**c**), γH2AX and 53BP1 foci (**d**), γH2AX foci (**e**) and pan-nuclear γH2AX (**f**) in NeuN^+^ L2/3 neurons. *n* = 5 (control) and *n* = 4 (MS). **g**, Schematic of the single-nucleus sequencing method. **h**, Pseudotime trajectory of DDR and apoptosis genes in MS L2/3ENs. *n* = 9 (control), *n* = 12 (MS). **i**, All DEGs (grey dots) overlaid with upregulated (red) or downregulated (blue) DDR DEGs in select cortical cell types. *n* = 9 (control), *n* = 12 (MS). **j**, Schematic of the disease course of DTA mice along weeks (W) after induction. **k**–**r**, Immunohistochemistry analysis of NeuN (**k**), CTIP2 (**l**), CUX1 (**n**,**o**), 53BP1, γH2AX and NeuN (**p**), OHdG and NeuN (**q**) and 8-OHG, 8-OHdG and 8-OHGua with NeuN (**r**), counterstained with DAPI (**k**,**l**) with cell counts (*n* = 5 per group (44 weeks), *n* = 3 per group (27 weeks)), PDDF or foci counts (*n* = 4 (5 weeks, control), *n* = 6 (5 weeks, DTA); *n* = 4 per group (10 weeks and 17 weeks)) and integrated density quantification (*n* = 4 mice per group; 8-OHdG: 127 control cells, 161 DTA cells; for 8-OHG, 8-OHdG, 8-OHGua, 100 cells per group). **m**, Chromogenic in situ hybridization for *Cux2* and *Cux2*^+^ cell counts in 44 week DTA mice. *n* = 4 (control) and *n* = 5 (DTA). Data are mean ± s.e.m. (**c**–**f** and **k**–**p**). The violin plots show the mean + quartiles (**p**–**r**). Statistical differences were determined using two-tailed unpaired *t*-tests (**c**–**f** and **m**–**p**), two-tailed Mann–Whitney *U*-tests (**q** and **r**) and one-way analysis of variance (ANOVA) with Šídák’s multiple-comparison test (**k**–**l** and **p**). Scale bars, 100 μm (**k**–**o**, **q** and **r**) and 10 μm (**b** and **p**). IN-VIP, vasoactive intestinal peptide-expressing interneurons. IntDens, integrated density. Source data

MS, an autoimmune disease targeting myelinating oligodendrocytes of the central nervous system (CNS), is the most common cause of neurological disability in young adults^8^. MS shows an initial relapsing–remitting course followed by progression, characterized by clinical deterioration and brain atrophy with upper cortical layer thinning^9^. Although the outcomes of individuals with relapsing–remitting MS have improved with therapies targeting T and B cells, patients still show disease progression and brain atrophy^10,11^, highlighting the need to better understand mechanisms of neurodegeneration in MS. Previously we reported selective loss of L2/3ENs in MS^2^, but whether this was indicative of their intrinsic vulnerability to direct neuroinflammatory injury remained unclear. L2/3ENs are marked by expression of CUT homeodomain proteins (such as CUX1, CUX2 and SATB2; Fig. 1a), which are known to promote DNA BER after oxidative stress^12^, yet their cortical DDR function remains largely unexplored. We previously reported significant upregulation of the genome stability factor non-coding RNA activated by DNA damage (NORAD) in MS L2/3ENs^2,13^, suggesting increased DNA damage burden. Here we investigated the mechanisms of vulnerability of L2/3ENs in relation to DNA damage and repair in the setting of neuroinflammatory stress.

## DNA damage burden in MS L2/3ENs

**Extended Data Fig. 1:**
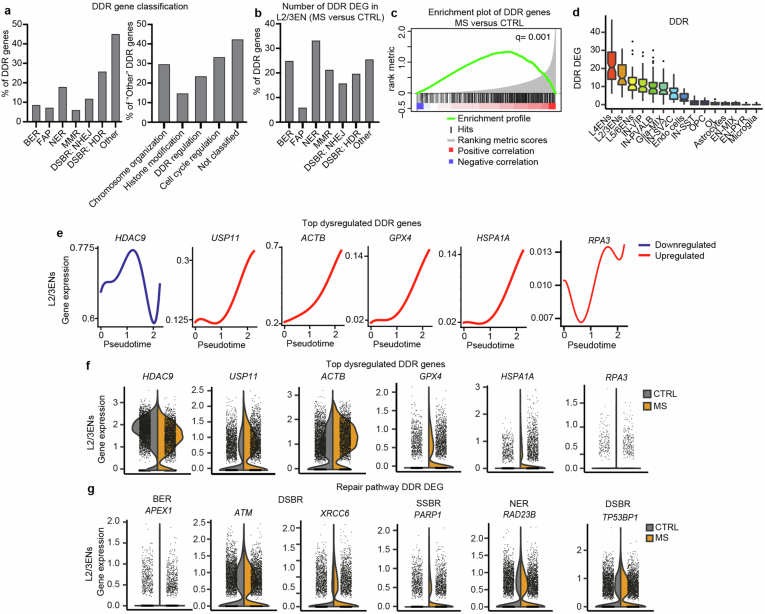
DDR dysregulation in MS cortical neurons. **a**, Classification of 698 DDR genes according to repair pathway (left) or other biological process mediating DDR (right). **b**, Classification of 159 DDR DEG in MS L2/3ENs according to DNA repair pathway (*n* = 12 MS, *n* = 9 CTRL). **c**, Geneset enrichment plot for DDR genes (*n* = 12 MS, *n* = 9 CTRL; FDR 0.05, log2 fold change 0.1). **d**, Box plot of DDR DEG across cell subtypes in human MS (*n* = 12 MS, *n* = 9 CTRL). Plot depicts median and interquartile range (IQR) of DEG calculated after downsampling (100 DEG analyses per cell cluster). **e**, Pseudotime trajectory (**e**) and bean plots (**f**) of top down (blue) and upregulated (red) DDR DEG in L2/3ENs (*n* = 12 MS, *n* = 9 CTRL). **g**, Bean expression plots of base excision repair (BER), double-strand break repair (DSBR), single-strand break repair (SSBR) and nucleotide excision repair (NER) factors in MS L2/3ENs (*n* = 12 MS, *n* = 9 CTRL). FAP = Fanconi Anaemia pathway, MMR = mismatch repair, NHEJ = non-homologous end joining, HDR = homology directed repair.

To investigate DNA damage in MS, we first performed immunohistochemistry to identify DNA damage marked by 53BP1 and γH2AX foci^14,15^ in cortical grey matter lesions from four cases of MS versus controls (Fig. 1b–f and Supplementary Table 1). As shown, MS L2/3ENs exhibited elevated γH2AX^+^ and 53BP1^+^γH2AX^+^ foci (Fig. 1d,e), and an increased proportion of neurons with pan-nuclear 53BP1 (Fig. 1c), despite no change in terminal cell death indicated by pan-nuclear γH2AX reactivity^16^ (Fig. 1f). MS layer 2, 3 and 4 excitatory neurons (L2–4ENs) showed heightened apoptosis gene expression and a robust pseudotime transcriptional DDR response (from a curated DDR list of 698 genes; >80% Gene Ontology (GO): 0006281: DNA repair; Extended Data Fig. 1a,b and Supplementary Tables 2 and 3); by contrast, these signatures were weak or absent in other cortical cell types (Fig. 1h,i and Extended Data Fig. 1c–g). Genes critical for chromatin remodelling (*GPX4*), BER and SSB repair (*APEX1*, *RPA3*, *PARP1* and* HSPA1A*), nucleotide-excision repair (NER; *ACTB*, *HSPA1A*, *RAD23B* and *RPA3*) and DSB repair (*ATM*, *RPA3*, *TP53BP1* and *XRCC6*; Extended Data Fig. 1e–g) were upregulated in pseudotime, suggesting activation of multiple repair pathways. These findings indicate elevated DNA damage burden despite DDR activation in vulnerable MS L2/3ENs.

## L2/3EN loss in MS mouse models

**Extended Data Fig. 2:**
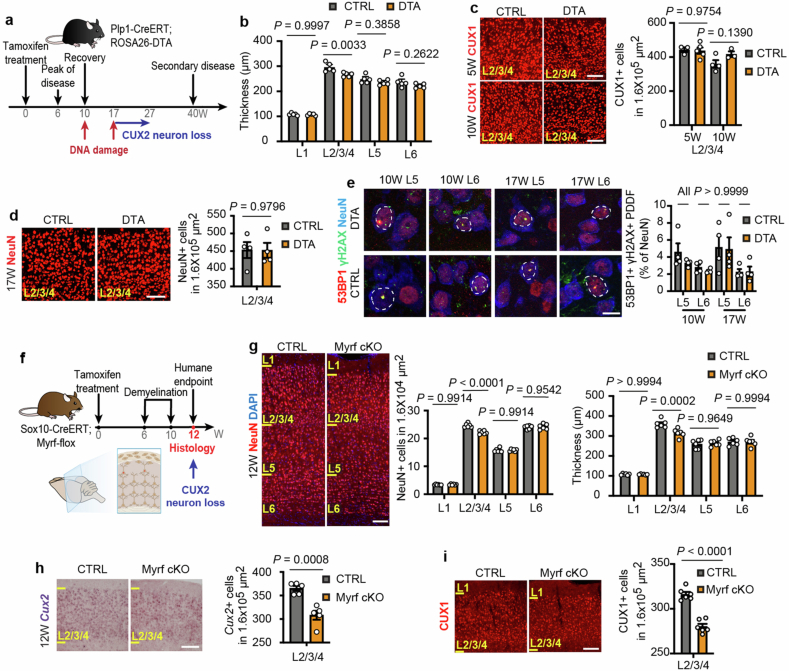
Specific loss of layer 2-4 in DTA and Myrf cKO mice. Schema of disease course of DTA mice (**a**) or Myrf cKO mice (**f**) with DNA damage and CUX2 neuron loss findings indicated. **b**, Quantification of cortical layer thickness from NeuN immunohistochemistry (Fig. 1k) at 44 W in DTA mice (*n* = 5 per group). Immunohistochemistry for CUX1 (**c**, **i**), NeuN (**d**), 53BP1, γH2AX and NeuN (**e**), NeuN and DAPI counterstain (**g**) in the primary somatosensory cortex of DTA and Myrf cKO mice, with CUX1+ cell counts (*n* = 3, 5 W CTRL and 10 W DTA, *n* = 4, 10 W CTRL, *n* = 5, 5 W DTA, *n* = 6 per group Myrf cKO), NeuN+ cell counts (*n* = 4 per group DTA, *n* = 6 per group Myrf cKO), PDDF+ neuron cell counts (*n* = 4 per group), and layer thickness quantification (n = 6 per group). (**h**) Chromogenic in situ hybridization for *Cux2* and *Cux2*+ cell counts in 12 W Myrf cKO mice (*n* = 5 per group). Error bars show mean +/− SEM and statistical differences were determined by one-way ANOVA with Holm-Šídák’s multiple comparisons tests (**b**, **c**, **g**), Kruskal-Wallis test with Dunn’s multiple comparisons test (**e**) or two-tailed unpaired t-tests (**d**, **h**, **i**). Scale bars = 100 μm or 10 μm (**e**). Source data

**Extended Data Fig. 3:**
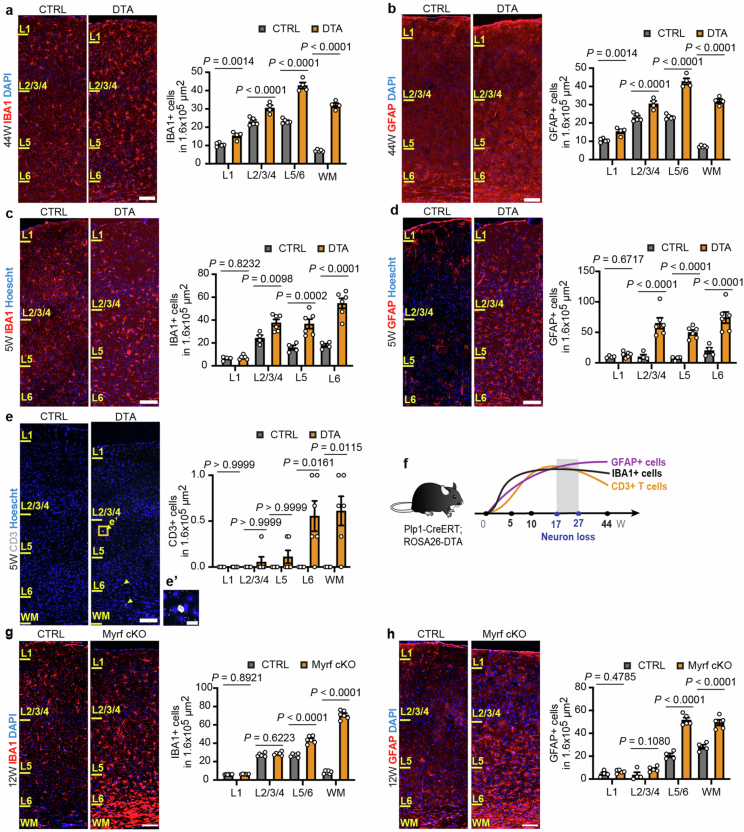
Neuroinflammation in DTA and Myrf cKO mice. Immunohistochemistry for IBA1 (**a**, **c**, **g**), GFAP (**b**, **d**, **h**), or CD3 (**e**), counterstained with DAPI (**a**–**b**, **g**–**h**) or Hoescht (**c-e**) in primary somatosensory cortex of 44 W DTA (**a**–**b**), 5 W DTA (**c-e**) or 12 W Myrf cKO (**g**–**h**) mice, with layer cell counts in DTA mice (*n* = 4 DTA 44 W, *n* = 5, 44 W DTA, *n* = 4, 5 W CTRL, *n* = 6, 5 W DTA) or Myrf cKO mice (*n* = 6 per group IBA1, *n* = 4 CTRL or *n* = 5 cKO GFAP). Error bars show mean +/− SEM and significant differences were determined with one-way ANOVA with Holm-Šídák’s multiple comparisons tests (**a**–**d**, **g**–**h**) or a Kruskal-Wallis test with post Dunn’s multiple comparison test (**e**). **f**, Summary schema of glial proliferation and T cell infiltration, incorporating results from Traka and colleagues^17^ in DTA mice. WM = white matter. Scale bars = 100 μm (**a**–**e**, **g**–**h**) or 10 μm (**e**’). Source data

We next investigated selective L2/3EN vulnerability in mouse models of pan-cortical demyelination and neuroinflammation. *Plp-creERT2 *×* ROSA26-DTA-STOP-floxed* mice (hereafter, DTA mice) exhibit widespread oligodendrocyte death shortly after DTA induction followed by a transient recovery phase and a subsequent secondary clinical decline associated with T cell infiltration^17^ (Fig. 1j). As shown in Fig. 1k–n and Extended Data Fig. 2b, at 44 weeks after tamoxifen induction (secondary disease), we found a >10% reduction in L2/3ENs marked by CUX1 and *Cux2* and cortical thinning in primary somatosensory cortex. By contrast, the numbers of CTIP2^+^ layer 5 and 6 (L5/L6) ENs were stable despite pan-cortical reactive gliosis and CD3^+^ T cell infiltration (Fig. 1l and Extended Data Fig. 3a–e). To determine when L2/3EN losses occurred, we performed sequential counts at 5, 10, 17 and 27 weeks. As shown (Fig. 1o and Extended Data Fig. 2c,d), L2/3EN loss occurred between 17 and 27 weeks, despite pan-cortical neuroinflammation and T cell infiltration being observed by 5 weeks (Extended Data Fig. 3c-e). L2/3ENs demonstrated increased DNA damage burden from 10 weeks (Fig. 1p), 5 weeks after the peak of demyelination^18^. DNA damage in DTA L2/3ENs included 53BP1^+^γH2AX^+^ persistent DNA damage foci^19^ (PDDF), DNA DSBs indicated by 53BP1^+^ foci, SSBs and DSBs indicated by γH2AX^+^ foci (Fig. 1p), and oxidative DNA and RNA damage (labelled by 8-hydroxyguanosine (8-OHG), 8-hydroxy-2′-deoxyguanosine (8-OHdG) and 8-hydroxyguanine (8-OHGua); Fig. 1q,r). By contrast, L5/6 projection neurons had normal levels of PDDF (Extended Data Fig. 2e). We next investigated L2/3 vulnerability in *Myrf*-conditional-knockout mice (*Myrf*-cKO), which results in failure of oligodendrocyte maturation, coupled with demyelination and neuroinflammation^20^. This model also showed L2/3 thinning and selective loss of L2/3ENs (Extended Data Fig. 2f–i), despite milder neuroinflammation in layers 1, 2, 3 and 4 (Extended Data Fig. 3g,h). These findings show that elevated DNA damage in CUX2^+^ L2–4 neurons is associated with their selective vulnerability to neurodegeneration during demyelination with neuroinflammation.

## L2/3EN DDR failure in MS mouse models

**Extended Data Fig. 4:**
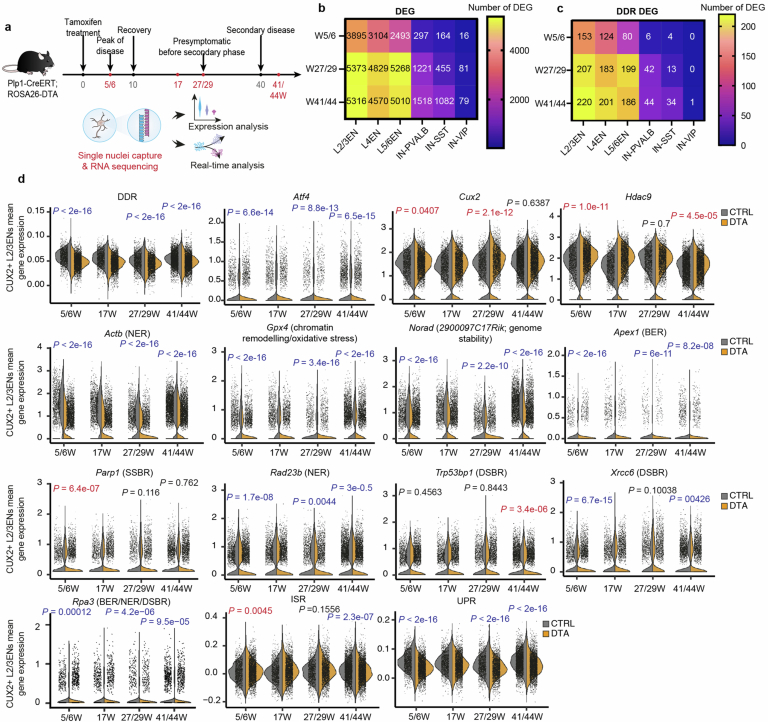
Longitudinal DEG analyses in DTA mice. **a**, Schema of disease course of DTA mice with indicated timepoints of single nuclei capture and RNA sequencing at weeks post DTA induction (W). Heatmap of DEG (**b**) and DDR DEG (**c**) and L2/3ENs mean expression (**d**) of DDR, integrated stress response (ISR) or unfolded protein response (UPR) genesets or individual genes across timepoints in DTA mice (*n* = 5 per group 5/6 W; *n* = 2 DTA and *n* = 5 CTRL 17 W, *n* = 3 per group 27/29 W and 41/44 W). Significantly DEGs (upregulated = red *P* values, downregulated = blue *P* values, not significantly regulated = black *P* values) were identified using Wilcoxon rank sum test with FDR 0.05 and log2 fold change 0.1. IN-PVALB = parvalbumin-expressing interneurons, IN-SST = somatostatin-expressing interneurons, IN-VIP = vasoactive intestinal peptide-expressing interneurons, NER = nucleotide excision repair, BER = base excision repair, SSBR = single-strand break repair, DSBR = double-strand break repair.

**Extended Data Fig. 5:**
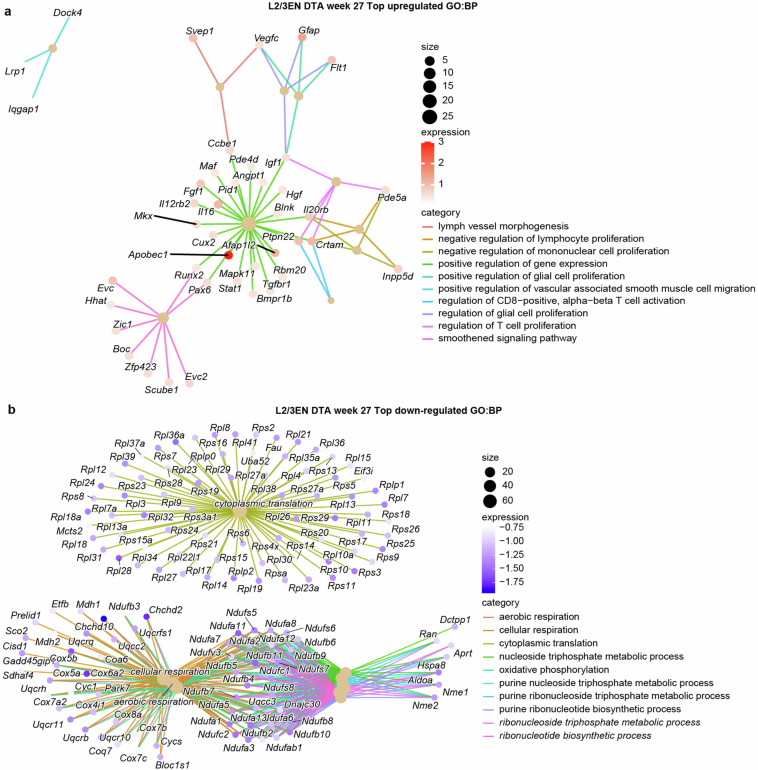
Pathway analysis of L2/3EN DEG in DTA mice. Netplot for significantly enriched biological process gene pathways in 27-week DTA mice (*n* = 3 per group), split by upregulated (**a**), or downregulated (**b**) genes. See also Supplementary Table 5. Significantly DEG were determined by Wilcoxon rank sum test with fdr 0.05 and log2 fold change 0.1. GO:BP = gene ontology: biological process.

To investigate gene dysregulation in L2/3ENs during demyelinating injury, we performed longitudinal single-nucleus RNA-sequencing (snRNA-seq) analysis of the DTA cerebral cortex from 5 to 44 weeks (Extended Data Fig. 4a). As in human MS, DTA L2/3ENs showed the most pronounced gene dysregulation, including DDR genes (Extended Data Fig. 4b,c and Supplementary Table 4). As shown in Extended Data Fig. 4d), longitudinal plots revealed significant downregulation of DDR genes in L2/3ENs during the disease course, including those for chromatin remodelling (*Gpx4*), BER and SSB repair (*Apex1*, *Parp1* and* Rpa3*), NER (*Actb*,* Rad23b* and* Rpa3*) and NHEJ repair (*Xrcc6*, also known as *Ku70*); *Atf4* was also significantly depleted, collectively suggesting vulnerability to DNA damage^21^. By contrast, *Cux2*, *Hdac9* and *Trp53bp1* (encoding 53BP1) were elevated, with *Trp53bp1* specifically elevated at 17 weeks and 41 or 44 (41/44) weeks, suggesting a requirement for DSB repair and genome stability. GO analysis indicated depression of cytoplasmic translation, respiration, mitochondrial respiratory chain complex assembly, purine nucleoside and ribonucleoside triphosphate metabolism and the unfolded protein response (Extended Data Fig. 5b and Supplementary Table 5). Moreover, the L2/3EN profile showed prominent dysregulation of neuroinflammation and immune signalling, such as glial cell and T cell proliferation and activation, and lymph vessel morphogenesis (Extended Data Fig. 5a). These results suggest that pan-cortical demyelination results in transcriptional repression and failure of the DDR in L2/3ENs before their degeneration.

## *Cux2* protects against postnatal injury

**Fig. 2:**
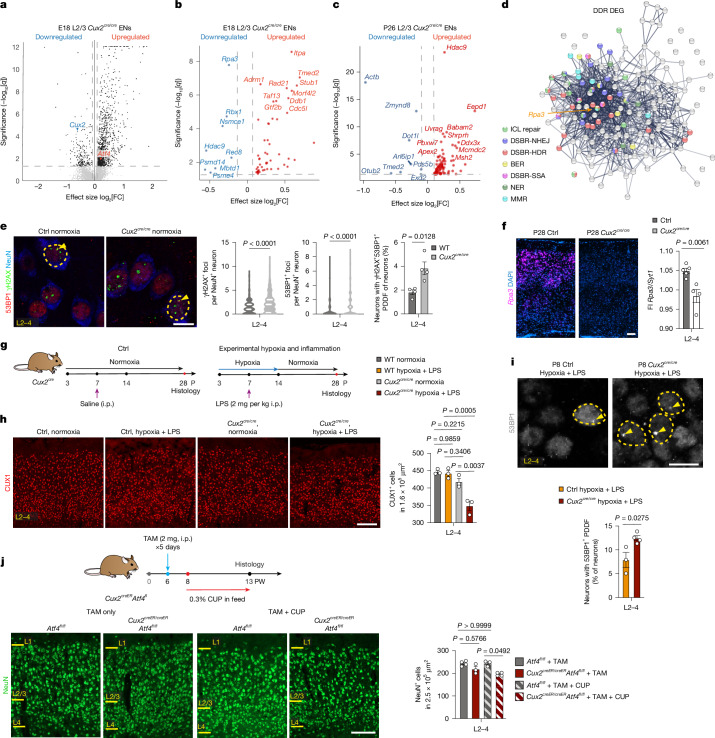
*Cux2* regulates the DDR and mediates L2/3EN resilience with *Atf4* against acute postnatal injury. **a**–**d**, Volcano plot of DEGs (**a**) DDR DEGs (E18 (**b**) and P26 (**c**)) and STRING plot (**d**)of combined DDR DEGs in L2/3ENs from *Cux2*^*cre/cre*^ versus control mice. *n* = 3 per group. The grey dotted lines indicate significance and fold change (FC) cut-offs at 0.05 and 0.1, respectively. The node colouring in the STRING plot depicts functional DDR annotation. **e**, Immunohistochemistry analysis of 53BP1, γH2AX and NeuN, and counts of NeuN^+^ cells with γH2AX^+^ foci, 53BP1^+^ foci and PDDF in L2–4ENs in *Cux2*^*cre*^ mice. *n* = 4 per group. **f**, RNAscope analysis of *Rpa3*,* Cux2* and *Syt1* with DAPI counterstain (*n* = 4 (control) and *n* = 5 (*Cux2*^*cre/cre*^) mice; Extended Data Fig. 6c), and fluorescence intensity (FI) quantification of *Rpa3* over *Syt1* in L2–4. **g**, Schematic of the experimental paradigm across postnatal days (P) for inducing hypoxic and neuroinflammatory stress in *Cux2*^*cre*^ mice. **h**,**i**, Immunohistochemistry analysis of CUX1 (**h**; *n* = 3 mice per group) or 53BP1 and NeuN (**i**; *n* = 3 (control) and *n* = 4 (*Cux2*^*cre/cre*^) mice) in L2–4 from *Cux2*^*cre/cre*^ and control mice under hypoxia and LPS with quantification. The yellow outlines depict cells with 53BP1^+^ foci (arrowheads). **j**, Schematic of tamoxifen (TAM) and acute cuprizone (CUP)-induced demyelination in *Cux2*^*creER*^*Atf4*^*fl*^ mice, and NeuN immunohistochemistry analysis in the primary somatosensory cortex of postnatal week (PW) 13 mice and quantification. *n* = 4 (*Atf4*^*fl/fl*^ + TAM and *Cux2*^*creER/ER*^*Atf4*^*fl/fl*^ + TAM + CUP) and *n* = 3 (*Atf4*^*fl/fl*^ + CUP + TAM and *Cux2*^*creER/ER*^*Atf4*^*fl/fl*^ + TAM). i.p., intraperitoneal. For **e** (left and middle), the violin plots show the mean + quartiles; significant differences were determined using two-tailed Mann–Whitney *U*-tests. For **e** (right), **f** and **h**–**j**, data are mean ± s.e.m.; significant differences were determined using two-tailed unpaired *t*-tests (**e**,** f** and** i**), one-way ANOVA with Tukey’s multiple-comparisons test (**h**) and Kruskal–Wallis tests with post Dunn’s multiple-comparison test (**j**). Scale bars, 100 μm (**f**,**h** and **j**) or 10 μm (**e** and** i**). ICL, interstrand cross-link; DSBR, DSB repair; HDR, homology-directed repair; SSA, single-strand annealing. Source data

**Extended Data Fig. 6:**
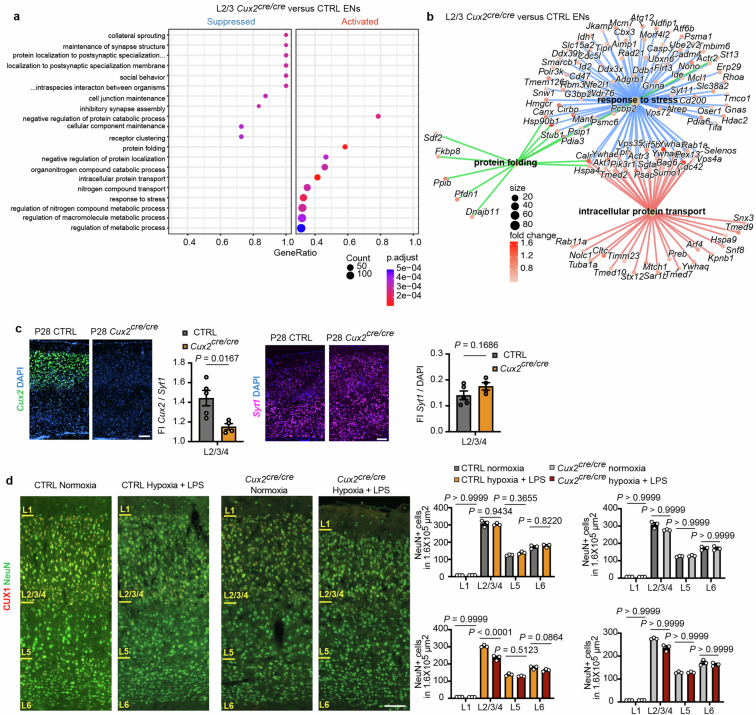
Pathway dysregulation and L2-4 selective vulnerability following *Cux2* loss and acute neuroinflammatory injury in mice. Dot plot (**a**) and netplot (**b**) of significantly enriched suppressed or activated pathways in E18 *Cux2*^*cr/cre*^ mice versus CTRL mice (*n* = 3 mice per group). Significant DEG were determined by Wilcoxon rank sum test with fdr 0.05 and log2 fold change 0.1 **c**, RNAscope for *Rpa3, Cux2* and *Syt1* with DAPI counterstain (*n* = 5 CTRL, *n* = 4 *Cux2*^*cre/cre*^ mice; see also Fig.2f), with fluorescence intensity quantification of *Cux2*/*Syt1* and *Syt1*/DAPI in cortical layer (L) 2-4. **d**, NeuN immunohistochemistry in *Cux2*^*cre/cre*^ and CTRL mice under normoxia or hypoxia and lipopolysaccharide (LPS) injection with quantification of NeuN+ cells per layer (*n* = 3 mice per group). Bars indicate mean +/− SEM with statistical differences tested via two-tailed unpaired t test (**c**), Kruskall-Wallis test with post Dunn’s multiple comparisons test (**d**: CTRL versus *Cux2*^*cre/cre*^ normoxia and *Cux2*^*cre/cre*^ normoxia versus hypoxia + LPS), or one-way ANOVA with Šídák’s multiple comparisons test (**d**: hypoxia + LPS CTRL versus *Cux2*^*cre/cre*^ and CTRL normoxia versus hypoxia + LPS). Scale bars = 100 μm. ENs = excitatory neurons. Source data

*CUX2* is an MS-risk-associated gene^22^ that is specifically expressed in cortical L2/3ENs, in which it might have roles in BER or other DDR regulation. To investigate this, we first performed snRNA-seq analysis of embryonic day 18.5 (E18.5) and postnatal day 26 (P26) cortices from *Cux2*^*cre*^ null mice (whereby Cre targeted to *Cux2* renders it null) and controls (Fig. 2a–c). As shown in Fig. 2b–d, Supplementary Tables 6–8 and Extended Data Fig. 6a,b, *Cux2*^*cre/cre*^ L2/3ENs demonstrated broad DDR gene dysregulation including upregulation of *Atf4* (Fig. 2a–c) and downregulation of the multifunctional RPA complex subunit *Rpa3*^23^. STRING analyses demonstrated RPA3 at the core of the dysregulated DDR network (Fig. 2d). We validated loss of *Rpa3* expression in the *Cux2*^*cre/cre*^ mouse cortex (Fig. 2f and Extended Data Fig. 6c). Dysregulated DDR signalling was associated with elevated baseline DNA damage in *Cux2*^*cre/cre*^ mice (Fig. 2e). Given these findings, we tested a *Cux2* resilience function after neonatal injury, at stages in which L2/3 axons are not yet myelinated. As shown (Fig. 2g,h and Extended Data Fig. 6c), acute neonatal hypoxic and inflammatory (lipopolysaccharide (LPS) induced) injury resulted in a significant decrease in L2/3ENs (CUX1^+^NeuN^+^ cells) in *Cux2*^*cre/cre*^ mice relative to wild-type (WT) or unchallenged *Cux2*^*cre/cre*^ mice. Indeed, hypoxia and LPS treatment of *Cux2*^*cre/cre*^ mice further elevated 53BP1^+^γH2AX^+^ PDDF in L2/3ENs (Fig. 2i). Thus, *Cux2* is essential to mitigate DNA damage, regulate the DDR and promote resilience of L2/3ENs against injury.

## *Cux2* and *Atf4* confer L2/3EN resilience

**Extended Data Fig. 7:**
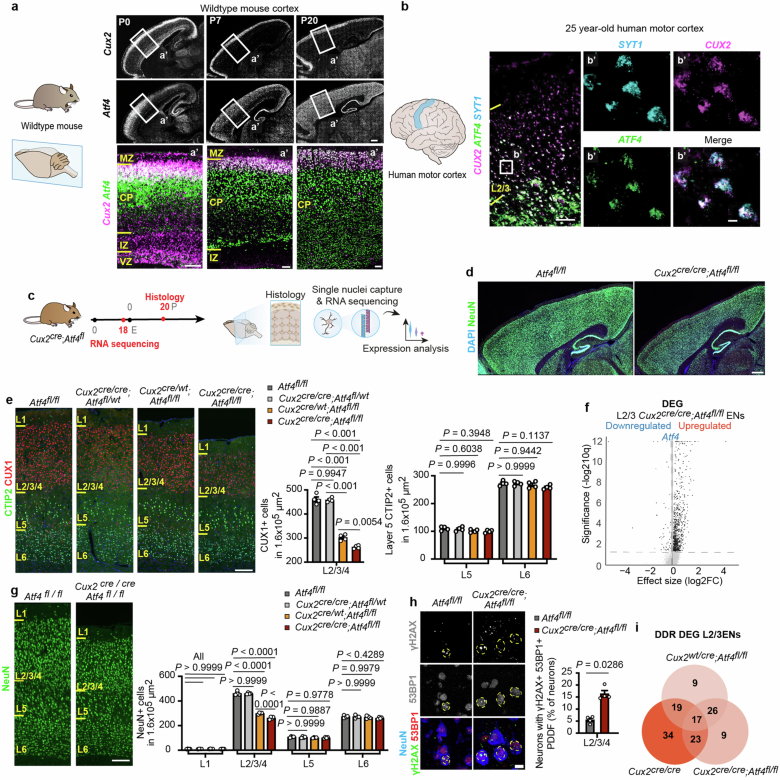
CUX2 and ATF4 collaborate to mediate viability of L2-4 neurons during development. **a**, RNAscope for *Cux2* and *Atf4* on postnatal day (P)0, P7 and P20 wildtype (*n* = 3 P0, *n* = 3 P20 mice, *n* = 5 P7 mice) sagittal brain sections with schema (left) and cortical plate (CP) inset (**a’**). **b**, Schema of human brain with motor cortex outlined in blue (left) and RNAscope for *CUX2, ATF4* and *SYT1* in 25-year-old motor cortex (right; *n* = 1). L2/3 is bordered by yellow lines and white box indicates region of insets (**b’**). **c**, Schema of embryonic day (E) or postnatal day (P) collection timepoints in *Cux2*^*cre*^*;Atf4*^*fl*^ mice. Immunohistochemistry for NeuN (**d**, **g**), CUX1 and CTIP2 (**e**) or γH2AX, 53BP1 and NeuN (**h**) with DAPI counterstain (**d**) on the primary somatosensory cortex of P20 *Cux2*^*cre*^*;Atf4*^*fl*^ mice, with quantification of L2-4 CUX1+ cells and L5/6 CTIP2+ cells (*n* = 4 per group), NeuN+ cells (*n* = 4 per group) and 53BP1 + /γH2AX + PDDF (*n* = 4 per group). **f**, Volcano plot of DEG in L2/3ENs from *Cux2*^*cre/cre*^*;Atf4*^*fl/fl*^ versus *Atf4*^*fl/fl*^ mice (*n* = 3 mice per group). Grey dotted lines indicate significance and fold change cutoffs at 0.05 and 0.1 respectively. **i**, Venn diagram of DDR DEG across genotypes from E18 *Cux2*^*cre*^*;Atf4*^*fl*^ mice (*n* = 3 per group; see also Supplementary Table 9). Significant DEG were determined by Wilcoxon rank sum test. Error bars show mean +/− SEM with significant differences determined by one-way ANOVA with Tukey’s multiple comparisons test (**e:CUX1**), one-way ANOVA with Šídák’s multiple comparisons test (**e:CTIP2, g**) or two-tailed Mann-Whitney test (**h**). Scale bars = 500 μm (**a**), 100 μm (**a’**, **b**, **d**–**g**), or 10 μm (**b’**, **h**). MZ = marginal zone, IZ = intermediate zone, VZ = ventricular zone. Source data

In a companion paper^21^ we found that *Atf4* has a crucial role in mitigating DNA damage in embryonic CUX2^+^ cortical progenitors. *Cux2* and *Atf4* continue to be co-expressed in postnatal L2/3ENs (Extended Data Fig. 7a), a pattern that is conserved in the human motor cortex (Extended Data Fig. 7b). As *Atf4* was upregulated in *Cux2*^*cre/cre*^ mice, we reasoned that ATF4 was recruited to compensate for *Cux2* loss in DDR. We crossed *Cux2*^*cre*^ mice to *Atf4*^*fl*^ mice to generate *Cux2/Atf4* double-cKO mice (Extended Data Fig. 7c), which revealed upper-layer cortical thinning and selective L2/3 CUX1^+^NeuN^+^ cell losses that were gene dose responsive (for example, *Cux2*^*cre/WT*^*Atf4*^*fl/fl*^ versus *Cux*^*cre/cre*^*Atf4*^*fl/fl*^; Extended Data Fig. 7d–g). Moreover, we observed upregulation of genes involved in the DDR (Extended Data Fig. 7f,i and Supplementary Table 9), accompanied by a threefold increase in 53BP1^+^γH2AX^+^ PDDF in L2/3ENs (Extended Data Fig. 7h) in *Cux*^*cre/cre*^*Atf4*^*fl/fl*^ mice, indicating essential functions for these genes in resilience and DDR regulation during development. Next, we examined whether *Cux2* and *Atf4* function is required for postnatal resilience against acute injury. We intercrossed inducible *Cux*^*creER*^ and *Atf4*^*fl*^ mice and subjected animals to cuprizone-induced demyelinating injury at 2 weeks after tamoxifen induction. As shown (Fig. 2j), tamoxifen administration alone did not significantly affect L2/3EN density, indicating that *Cux2* and *Atf4* function is not required for maintaining their viability in normal (non-injury) postnatal conditions. By contrast, after cuprizone-induced demyelination, we observed significant loss of NeuN^+^ L2/3 neurons in *Cux2*^*creER/creER*^*Atf4*^*fl/fl*^ mice (Fig. 2j), establishing that *Cux2* and *Atf4* function is required for postnatal L2/3 neuron resilience against cuprizone-induced demyelinating injury.

## *CUX2* and *ATF4* repair DNA damage

**Fig. 3:**
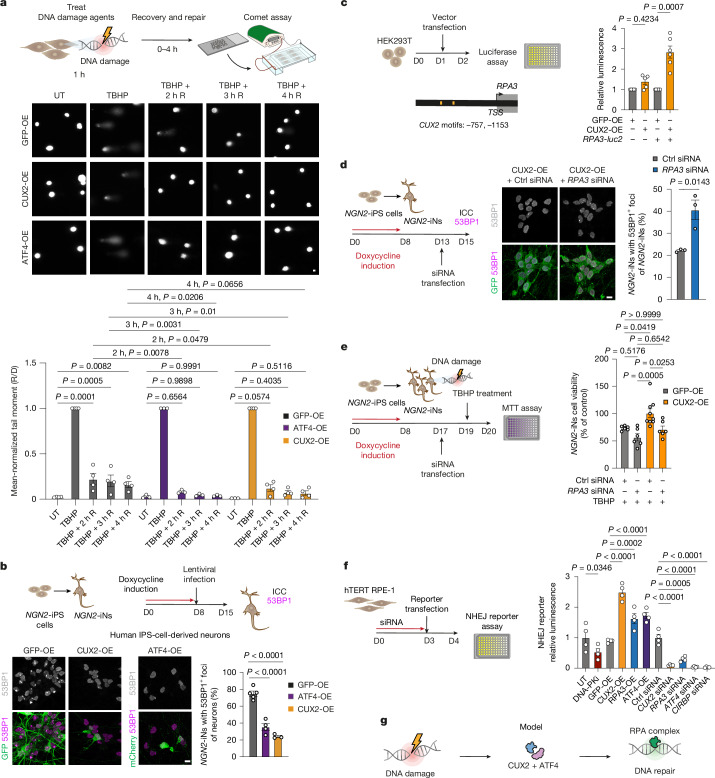
CUX2 and ATF4 promote DNA DSB repair. **a**, Schematic of the alkaline comet assay(top) and representative SYBR-Green-stained images (middle), assessing DNA damage (D) and recovery (R) over 0–4 h in SH-SY5Y cells with GFP, CUX2 or ATF4 overexpression (OE), either untreated (UT) or acutely treated with TBHP (200 µM, 1 h). Quantification shows the recovery-to-damage (R/D) tail moment ratios. *n* = 3 (ATF4-OE groups and CUX2-OE UT) and *n* = 4 (other groups). **b**, *NGN2*-iPS cell differentiation into *NGN2*-iNs followed by GFP, CUX2 or ATF4 overexpression. Immunocytochemistry analysis of GFP or mCherry and 53BP1 with quantification of 53BP1^+^ foci. *n* = 5 (GFP), *n* = 3 (CUX2) and *n* = 4 (ATF4). **c**, Promoter luciferase reporter assays in HEK293T cells at day (D) 2 after plating, 24 h after vector transfection. *luc2* (*n* = 5) and *RPA3*-*luc2* (*n* = 6). **d**, Experimental schematic and 53BP1 immunocytochemistry in CUX2-OE *NGN2*-iNs with control or *RPA3* siRNA treatment. *n* = 3. **e**, MTT viability assays after siRNA or TBHP treatment in GFP- or CUX2-OE *NGN2*-iNs. *n* = 6 (GFP-OE and CUX2-OE + *RPA3*-siRNA groups) and *n* = 9 (CUX2-OE + control siRNA). **f**, NHEJ reporter assays in GFP-, CUX2-, ATF4- or CIRBP-overexpressing hTERT RPE-1 cells or WT cells treated with siRNA (on day 0) or 1 μM AZD7648 DNA-PK inhibitor (DNA-PKi; on day 3) with quantification of NanoLuc luciferase activity normalized to the internal firefly luciferase control (*n* = 4). **g**, Schematic of a model in which CUX2 and ATF4 proteins repair damaged DNA. Data are mean ± s.e.m. Statistical differences were determined using two-way ANOVA with Dunnett’s multiple-comparison test (**a**), one-way ANOVA with Šídák’s multiple-comparison test (**b**,** e** and **f**), Kruskal–Wallis test with Dunn’s multiple-comparison test (**c**) or two-tailed unpaired *t*-tests (**d**). Scale bars, 10 µm (**a**, **b** and **d**). Source data

**Extended Data Fig. 8:**
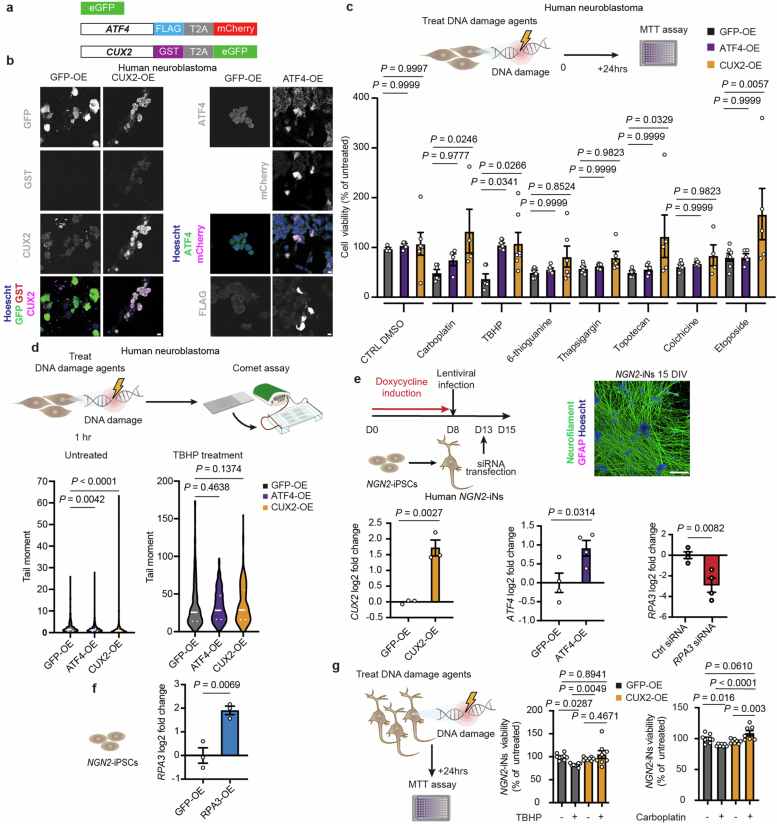
CUX2 and ATF4 regulate DNA repair kinetics. **a**, Schema of vector domains used for lentiviral overexpression (OE) of GFP, ATF4 and CUX2. **b**, Immunocytochemistry for CUX2, GST, GFP (left), or FLAG, ATF4, mCherry (right), counterstained with Hoechst in GFP-, CUX2-, or ATF4-OE SHSY5Y neuroblastoma cells. **c**, MTT viability assay in SHSY5Y human neuroblastoma cells with GFP-OE, CUX2-OE or ATF4-OE subjected to DMSO only (0.1%), Carboplatin (10μM), TBHP (200μM), 6-thioguanine (100μM), Thapsigargin (1μM), Topotecan (1μM), Colchicine (1μM), Etoposide (1μM) for 24 hrs, with schema of experimental design. Data was averaged from 3 technical replicates per trial (*n* = 4: CUX2+Carboplatin, CUX2+Colchicine; *n* = 5: Control/ATF4 + DMSO; CUX2+Thapsigargin, CUX2/ATF4+Topotecan, ATF4 +Colchicine, CUX2+etoposide; *n* = 6: CUX2 + DMSO, Control+Carboplatin, CUX2/ATF4 + 6-thioguanine, ATF4+thapsigargin; *n* = 7: remaining groups) **d**, Alkaline comet assay for DNA damage in SHSY5Y human neuroblastoma cells with GFP-OE, CUX2-OE or ATF4-OE untreated (UT) or subjected to TBHP (200 μM) for 1 hr with schema and quantification (*n* = 4). **e**, Neurofilament and GFAP immunocytochemistry and Hoescht counterstain (right) or quantification of *CUX2, ATF4* or *RPA3* transcripts in *NGN2*-iNs at 15 days in vitro (DIV; bottom, *n* = 3 per group *CUX2*, *n* = 4 per group *ATF4/RPA3*), with schema of differentiation course. **f**, Quantification of *RPA3* transcripts in *NGN2*-iPSCs with GFP-OE or RPA3-OE (*n* = 3 per group). **g**, MTT viability assays in *NGN2-*iNs with GFP-OE or CUX2-OE subjected to TBHP (200μM), or Carboplatin (10μM) at D18 for 24 hrs. Error bars show mean +/− SEM. Violin plots show median plus quartiles. Statistical differences were determined by one-way ANOVA with Holm-Šídák’s multiple comparisons test (**c**), Kruskal-Wallis test with post Dunn’s multiple comparisons test (**d**), two-tailed unpaired t tests (**e**–**f**) or one-way ANOVA with Šídák’s multiple comparisons test (**g**). Scale bars = 10 μm. Source data

The findings above were consistent with roles for *Cux2* and *Atf4* in prevention and/or repair of DNA damage. To discriminate between these possibilities, we first performed a chemical screen in SHSY5Y human neuroblastoma cells, distinguished by native expression of CUX2^24^. We faithfully induced overexpression of tagged human *CUX2* and *ATF4* using lentivirus and screened for viability in the presence of a variety of DNA damaging agents (Extended Data Fig. 8a–c). All agents induced SHSY5Y cytotoxicity, but CUX2 overexpression rescued cell death induced by carboplatin (DNA cross-linking), topotecan (DNA DSBs), etoposide (DNA DSBs) and *tert*-butyl hydroperoxide (TBHP, oxidative damage). However, ATF4 overexpression mitigated only oxidative TBHP-induced DNA damage. An alkaline comet assay with a recovery time course after TBHP treatment showed that *CUX2* and *ATF4* accelerate DNA repair (Fig. 3a,b). *CUX2* and *ATF4* overexpression conferred reduced DNA damage at the baseline, suggesting that they promote genomic integrity under homeostatic conditions (Extended Data Fig. 8d). While TBHP treatment induced similar levels of DNA damage (Extended Data Fig. 8d), overexpression of *CUX2* and *ATF4* facilitated complete repair of DNA damage 2 h after treatment versus the controls (GFP overexpression), which showed elevated DNA damage 4 h after treatment (Fig. 3a). To extend these findings to postmitotic human neurons (*NGN2*-iNs), neurogenin-2 (*NGN2*) expression was induced in pluripotent stem cells with doxycycline (*NGN2*-iPS cells; Fig. 3b). Lentiviral overexpression of *CUX2* and *ATF4* (Extended Data Fig. 8f) during *NGN2*-iNs differentiation significantly reduced baseline 53BP1^+^ DNA DSBs compared with the GFP controls (Fig. 3b). Furthermore, we established a *CUX2*-overexpression line of *NGN2*-iNs and found that this rescued cell losses in response to TBHP and carboplatin (Extended Data Fig. 8g). These results indicate that CUX2 and ATF4 act not to prevent but, rather, to repair DNA damage.

We next investigated whether CUX2 transcriptionally regulates *RPA3*, which showed reduced expression in *Cux2*^*cre/cre*^ mice and dysregulation in DTA mice and human MS (Fig. 2b,d,f and Extended Data Figs. 1d,e and 4d). Luciferase assays indicate that CUX2 can bind to the human *RPA3* promoter and activate downstream transcription (Fig. 3c). *RPA3* knockdown (Extended Data Fig. 8f) elevated DNA DSB burden in CUX2-overexpressing *NGN2*-iNs (Fig. 3d) and abolished the rescue of TBHP-induced neuronal death by CUX2 (Fig. 3e). Moreover, a NHEJ repair reporter assay demonstrated that CUX2, ATF4 and their respective transcriptional targets *RPA3* and *CIRBP*^21^ are essential for efficient and accurate NHEJ, the primary DNA DSB repair pathway of neurons (Fig. 3f). Together, these data demonstrate roles for CUX2 and ATF4 in DNA DSB repair (Fig. 3g).

## IFNγ induces selective loss of L2/3ENs

**Fig. 4:**
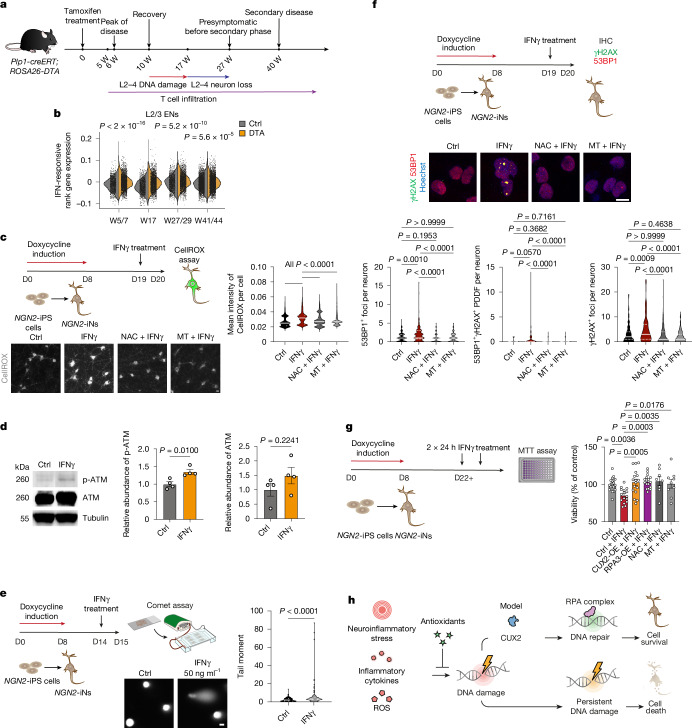
IFNγ induces oxidative DNA damage in human neurons. **a**, Disease course and pathological findings across weeks after induction in DTA mice. **b**, Split violin plot of IFN-responsive rank gene expression in L2/3ENs in DTA mice. *n* = 5 per group (5/6 weeks); *n* = 2 (DTA) and *n* = 5 (control, 17 weeks), *n* = 3 per group (27/29 weeks and 41/44 weeks). **c**, *NGN2*-iPS cell differentiation into *NGN2*-iNs followed by NAC (10μM) and MT (1 μM) pretreatment (1 h) before treatment with human IFNγ (50 ng ml^−1^, D19) and quantification of CellROX. Four thousand four hundred and seventy-three (control), 3,249 (control + IFNγ), 4,247 (NAC + IFNγ) and 3,801 (MT + IFNγ) cells from *n* = 4 independent differentiations per group. **d**, Western blot and quantification of ATM and phosphorylated ATM (p-ATM) normalized to tubulin in *NGN2*-iNs treated with IFNγ for 3 h. *n* = 4 independent differentiations per group. See Supplementary Fig. 1. **e**, Alkaline comet assay in IFNγ-treated (50 ng ml^−1^, 24 h) *NGN2*-iNs with quantification. One hundred and sixteen (control) and 108 (IFNγ) cells; *n* = 3 independent differentiations per group. **f**, Experimental design schematic, γH2AX/53BP1 immunocytochemistry and quantification of *NGN2*-iNs with DNA damage foci. Cells were pretreated with NAC (10 µM) or MT (1 µM) for 1 h, then exposed to IFNγ (50 ng ml^−1^) for 24 h. One hundred and forty-nine (control), 194 (IFNγ), 219 (NAC + IFNγ), 189 (MT + IFNγ) cells; *n* = 3 independent differentiations per group. IHC, immunohistochemistry. **g**, *NGN2*-iNs were pretreated with NAC (10 μM) and MT (1 μM) for 1 h before IFNγ treatment (50 ng ml^−1^). Viability was assessed using the MTT assay. *n* = 20 (control), *n* = 16 (control + IFNγ), *n* = 17 (CUX2-OE/RPA3-OE + IFNγ), *n* = 8 (NAC + IFNγ), *n* = 9 (MT + IFNγ) independent differentiations. **h**, The proposed model of neuroinflammatory DNA-damage-mediated cell death resolved by antioxidants or CUX2/ATF4-dependent repair. Data are mean ± s.e.m. (**d** and **g**). The violin plots show the median and quartiles (**b**,**c**,**e** and **f**). Significant differences were determined using Wilcoxon rank-sum tests (**b**), Kruskal–Wallis tests with post hoc Dunn’s multiple-comparison test (**c** and** f**), two-tailed unpaired *t*-tests (**d**), two-tailed Mann–Whitney *U*-tests (**e**) and one-way ANOVA with Šídák’s multiple-comparison test (**g**). For **c**, **e** and **f**, scale bars, 10 μm. Source data

**Extended Data Fig. 9:**
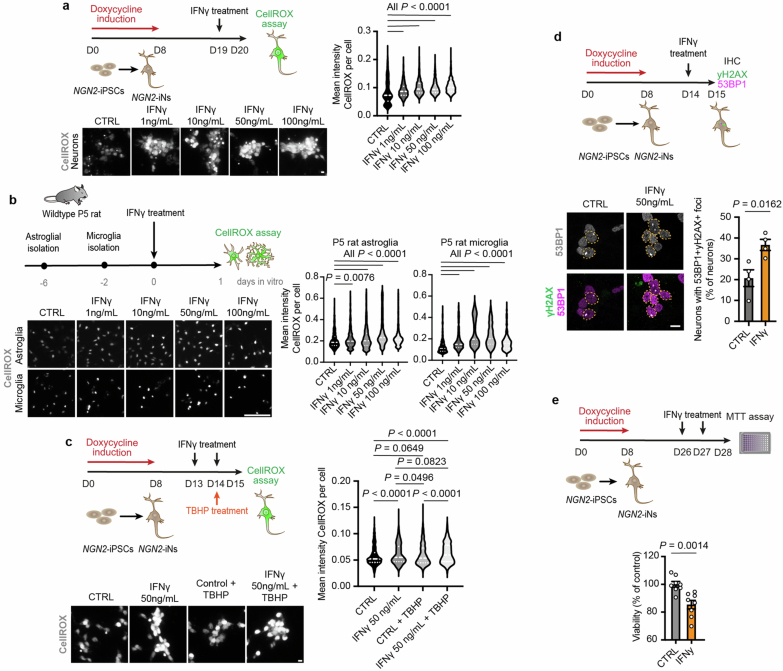
IFNγ induces oxidative stress and DNA damage in cortical cells. **a**, *NGN2*-iPSC differentiation into *NGN2*-iNs followed by IFNγ treatment at D19 and quantification of CellROX oxidative stress (1157 CTRL cells, 1161 1 ng/mL, 1374 10 ng/mL, 1191 50 ng/mL, 1295 100 ng/mL IFNγ-treated cells from *n* = 4 trials). **b**, Primary rat astroglia and microglia treated with IFNγ for 24hrs followed by CellROX assay with schema and quantification (astroglia: 952 CTRL cells, 1638 1 ng/mL, 1499 10 ng/mL, 1174 50 ng/mL, and 1947 100 ng/mL IFNγ-treated cells; microglia: 626 CTRL cells, 514 1 ng/mL, 552 10 ng/mL, 486 50 ng/mL, and 597 100 ng/mL IFNγ-treated cells from *n* = 4). **c**, Schema depicting treatment of *NGN2*-iNs with human IFNγ at 13 and 14D with TBHP treatment (20 μM) at 14D and quantification of CellROX (1150 CTRL, 1179 IFNγ, 1177 CTRL + TBHP, and 1260 IFNγ + TBHP cells from *n* = 4 trials). **d**, Immunocytochemistry for γH2AX and 53BP1 following treatment with 50 ng/mL IFNγ at D14 for 24hrs with schema and quantification of number of *NGN2*-iNs with γH2AX + /53BP1+ foci (*n* = 4). **e**, Experimental induction of *NGN2*-iNs, treatment with IFNγ (50 ng/mL) for 48 hrs with schema and quantification of viability via MTT (*n* = 8). Violin plots depict median plus quartiles. Error bars show mean +/− SEM. Statistical differences were determined by Kruskal-Wallis tests with post Dunn’s multiple comparisons tests (**a**–**c**) or two-tailed unpaired t tests (**d**–**e**). Scale bars = 100 μm (**b**) or 10 μm (**a**, **c**–**d**). Source data

We next focused on potential upstream mechanisms involved in inflammatory loss of L2/3ENs. First, we noted that the timing of DNA damage and DDR exhaustion in the DTA model was associated with cortical infiltration of T cells (Fig. 4a), which is known to cause cytotoxic injury in MS^3,4^, and to release inflammatory cytokines that stimulate ROS, including interferon-γ (IFNγ)^25–27^, which can bind to receptors that are widely expressed by resident brain cells^28^. In DTA mice, L2/3ENs showed significantly increased IFN-responsive gene expression across all timepoints (Fig. 4b). To determine whether IFNγ directly damages neurons and glia, we tested IFNγ-induced ROS production in mature *NGN2*-iNs, rat primary microglia or astrocytes. After 24 h of IFNγ treatment, all of the cell types displayed dose-dependent increases in ROS as measured by reaction with the fluorescent CellROX probe (Extended Data Fig. 9a,b). Fifty ng ml^−1^ IFNγ was sufficient to increase ROS in *NGN2*-iNs, which was exacerbated by mild TBHP treatment (20 μM; Extended Data Fig. 9c). In agreement with a cellular response to oxidative stress, we observed elevated phosphorylated ATM at Ser1981 in IFNγ-treated *NGN2*-iNs (Fig. 4d). ROS can cause oxidative DNA damage, and we found that IFNγ treatment significantly elevated basal DNA damage in *NGN2*-iNs as measured by the alkaline comet assay (Fig. 4e) and 53BP1^+^γH2AX^+^ foci (Fig. 4f and Extended Data Fig. 9d). DNA DSBs can form from unrepaired oxidative DNA lesions^29^, and we found that 53BP1^+^γH2AX^+^ DNA DSBs and PDDF formation were mitigated by pretreatment with the antioxidants *N*-acetylcysteine (NAC) or Mito-TEMPO (MT; Fig. 4c,f). Lastly, we observed that 50 ng ml^−1^ IFNγ reduced the viability of mature *NGN2*-iNs by approximately 15% (Extended Data Fig. 9e), while antioxidant treatment, or elevated DNA repair by CUX2 or RPA3 overexpression, provided resilience to cell death (Fig. 4g). These results indicate IFNγ directly induces ROS- and DNA-damage-associated cell death in human neurons that is diminished by treatment with antioxidants or elevated DNA repair.

**Fig. 5:**
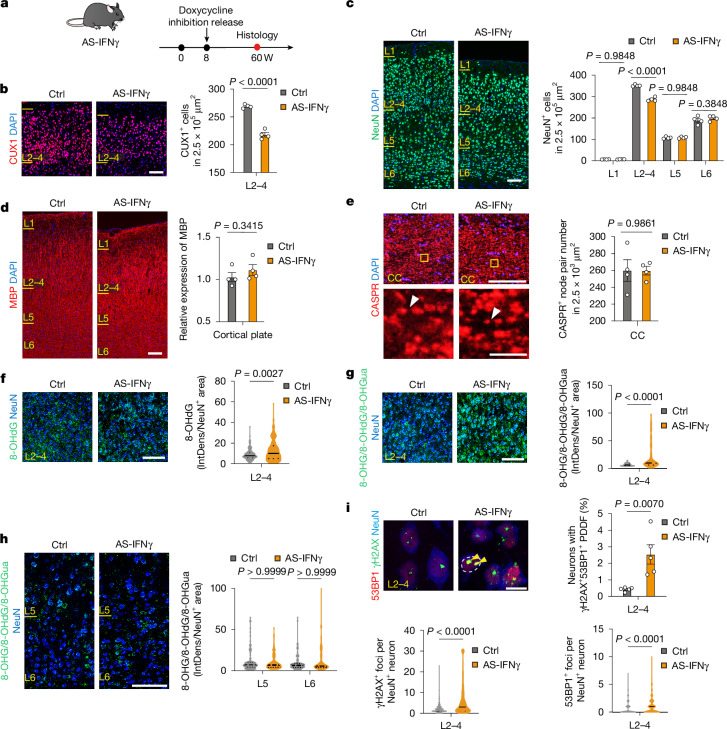
IFNγ induces oxidative DNA damage and selective L2/3 cortical neuron loss in mice. **a**, Schematic of doxycycline-inhibited astroglial IFNγ expression in AS-IFNγ mice and collection at 60 weeks. **b**–**e**, Immunohistochemistry analysis of CUX1 (**b**), NeuN (**c**) MBP (**d**) or CASPR (**e**) with DAPI counterstaining in AS-IFNγ mice (*n* = 4 per group), with layer (L) cell counts (**b**,**c**), relative expression (**d**) or CASPR^+^ node pair quantification (**e**). **f**–**h**, Immunohistochemistry analysis of 8-OHdG (**f**) or 8-OHG, 8-OHdG and 8-OHGua (**g**,**h**) and NeuN in L2–4 (**f**,**g**) or L5/6 (**h**); quantification of integrated density was normalized to the NeuN^+^ cell area measured. 8-OHdG: 140 (L2–4 control), 242 (L2–4 AS-IFNγ) cells from *n* = 5 mice per group; 8-OHG, 8-OHdG and 8-OHGua: 100 cells per group (L2–4), 100 (L5 control), 100 (L6 control), 101 (L5 AS-IFNγ) and 101 (L6 AS-IFNγ) cells from *n* = 4 mice per group. **i**, 53BP1, γH2AX and NeuN immunohistochemistry and counts of NeuN^+^ cells with foci and PDDF in L2–4 of AS-IFNγ mice. Seven hundred and six (control) and 553 (AS-IFNγ) cells from *n* = 5 mice per group. Data are mean ± s.e.m. Violin plots show the median and quartiles. Significant differences were determined using Holm–Šídák’s multiple-comparison test (**c**), two-tailed unpaired *t*-tests (**b**,**d**,**e** and **i** (PDDF only)), two-tailed Mann–Whitney *U*-tests (**f**,**g** and **i** (γH2AX^+^ foci and 53BP1^+^ foci)) and Kruskal–Wallis tests with Dunn’s multiple-comparison test (**h**). Scale bars, 100 μm (**b**–**h**) and 10 μm (**i**). CC, corpus callosum. Source data

**Extended Data Fig. 10:**
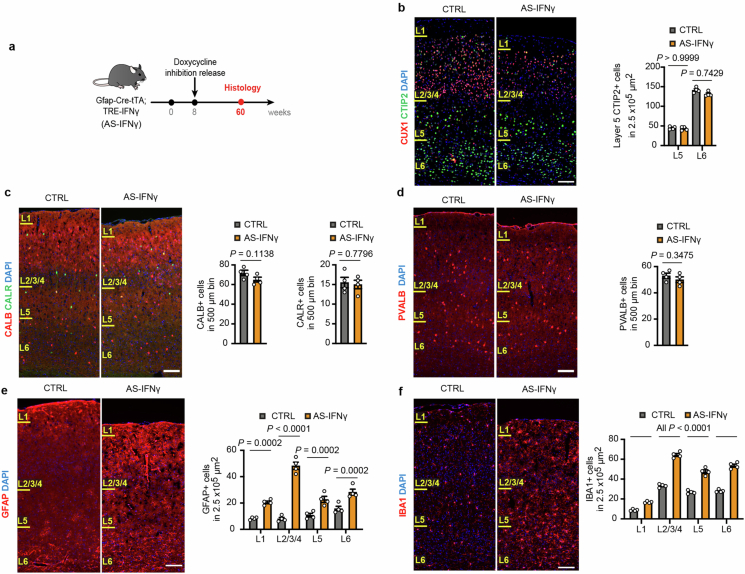
Deep layer and interneuron viability following ectopic conditional astroglial expression of IFNγ in mice. **a**, Schema of experimental ectopic and conditional doxycycline-inhibited IFNγ expression in astrocytes in *Gfap*-*tTA*;*TRE*-*IFNg* mice (AS-IFNγ) and collection timepoint at 60 weeks post doxycycline inhibition release. Immunohistochemistry for CUX1 and CTIP2 (**b**), Calbindin (CALB) and Calretinin (CALR; **c**), Parvalbumin (PVALB; **d**), GFAP (**e**), or IBA1 (**f**) counterstained with DAPI in AS-IFNγ mice with quantification of positive cells (*n* = 4 mice per group). Error bars show mean +/− SEM and statistical differences were determined by Kruskall-Wallis test with Dunn’s multiple comparisons test (**b**), two-tailed unpaired t tests (**c**–**d**) or one-way ANOVA with Holm-Šídák’s multiple comparisons tests (**e**–**f**). Scale bars = 100 μm. Source data

To investigate IFNγ effects in vivo, we used *Gfap-tTA;TRE-Ifng* mice (hereafter, AS-IFNγ mice), which ectopically express IFNγ from cortical astrocytes in a doxycycline-dependent manner (Fig. 5a). As shown in Fig. 5b,c, we observed a selective 18% decrease in CUX1^+^NeuN^+^ L2/3 neurons compared with the controls, 52 weeks after postnatal doxycycline release. By contrast, deep-layer projection neurons (NeuN^+^, CTIP2^+^) and interneuron populations (CALB1^+^, CALB2^+^, PVALB^+^) were unaffected (Fig. 5b and Extended Data Fig. 10a–d). Ectopic IFNγ expression caused pan-cortical gliosis as GFAP^+^ and IBA1^+^ cells were increased in number (Extended Data Fig. 10e,f). However, in contrast to the DTA and *Myrf-*cKO models, the significant loss of L2/3ENs in this model was not accompanied by gross demyelination in cortex (Fig. 5d) or disruption of internodes along the axons of interhemispheric projections (Fig. 5e), as assessed by the markers MBP and CNTNAP1 (also known as CASPR), respectively. In agreement with direct effects of IFNγ in neuronal cultures, we observed elevated oxidative RNA and DNA damage and 53BP1^+^γH2AX^+^ strand breaks in cortical L2–4 of AS-IFNγ mice (Fig. 5f,g,i). By contrast, L5/6 neurons did not show increased oxidative DNA and RNA damage (Fig. 5h). These findings suggest that IFNγ exposure results in DNA damage and L2/3EN neurodegeneration.

## Discussion

In MS, disproportionate thinning of upper cortical layers has been attributed to superficial leptomeningeal neuroinflammatory nodules that often contain T and B cells^9^ and losses of cortical excitatory neurons^2^ or interneurons^30^. Our findings indicate that DNA damage burden and inadequate repair in L2/3ENs during neuroinflammation accounts in part for their selective loss.

### Impaired DDR in L2/3ENs

CNS regional vulnerability has been linked to neurotrophin dependence, energy demand, reduced antioxidant capacity, oxidative stress and neuroinflammatory processes^31,32^. Consistent with findings of oxidative stress and DNA damage in the whole MS cortex^33–35^, and elevated somatic single-nucleotide variants in MS lesion-resident neurons^36^, we found that L2/3ENs in MS showed heightened DNA damage and activation of the DDR. We observed that cortical thinning and selective loss of L2/3 neurons were highly reproducible in several mouse models of pan-cortical demyelination and neuroinflammation (Supplementary Table 10). Transcriptomic analysis of DDR in the MS cortex showed that L2/3ENs were the most dysregulated, while neighbouring cell types were largely unaffected. Our findings of severe transcriptional dysregulation in L2/3ENs could be explained by decayed regulatory programs or epigenome erosion caused by DNA damage^37^, which may further dysregulate the DDR. Indeed, transcriptomic profiling in the DTA model revealed severely reduced expression of DDR genes in L2/3ENs including resilience factors (such as *Atf4*,* Rpa3* and *Norad*), at times preceding neuronal loss. In contrast to this real-time DDR exhaustion pattern in the mouse model, pseudotime patterns in human MS grey matter samples showed positive inflection. In addition to sampling and analytical differences between the mouse model and MS, the difference is probably because the profile of DDR exhaustion is under-represented in post-mortem MS tissue due to the protracted disease course where premorbid neurons evolve asynchronously and are scarce at any single time, with most L2/3 neurons attempting to upregulate the DDR in the face of genotoxic stress.

### CUX2 and ATF4 promote DNA repair

While CUX2 can enhance BER through protein–protein interactions in rat primary neurons^38^, such functions in vivo were unclear. This study shows CUX2 is a multifunctional DDR factor that promotes resilience to multiple types of DNA damage including enhancing NHEJ in response to DNA DSBs. Furthermore, we found that CUX2 activates *RPA3* in neurons as part of the DDR^23^. Seizures can arise from DNA damage accumulation^39^, which could partly explain why CUX2 dysfunction in mice and humans is associated with seizure susceptibility^40,41^. In the embryonic CNS, *Atf4* mitigated DNA damage through transcriptional control of factors involved in DSB repair and prevented p53-mediated cell death, specifically in CUX2 lineages in the cortex^21^. We were able to demonstrate a conserved function for ATF4 and target CIRBP in promoting DSB repair through NHEJ, which is likely to underlie the enhanced resilience function of ATF4 in the postnatal cortex. Together our findings implicate pathways converging on NHEJ-mediated DSB repair as a therapeutic target for enhancing resilience of L2/3 cortical neurons.

### Selective sensitivity of L2/3ENs

Loss of trophic and axonal support by oligodendrocytes has been shown to contribute to oxidative damage and selective L2/3 neurodegeneration in mouse models^42^. Such functions are disrupted in MS and are thought to result in secondary axonal dying back and attrition of the neuron cell body^43^. Alternative proposals for neurodegeneration have included primary inflammatory and autoimmune-mediated damage to neurons^44^. Irrespective of the primary or secondary mechanisms, this study clearly demonstrates the intrinsic vulnerability of CUX2^+^ L2/3ENs in the setting of general demyelination and neuroinflammation. Moreover, several lines of evidence suggest primary degenerative mechanisms in L2/3: first, a study reported loss of CUX2 neurons within the boundaries of MS cortical lesions^2^, consistent with direct impact to the cell soma of L2/3ENs. Second, we observed in the DTA model that neurodegeneration did not occur acutely with demyelination and oligodendrocyte loss but, rather, months later, accompanied by elevated DNA damage and a secondary wave of T cell infiltration, a source of IFNγ^25–27^. Third, we found direct toxic effects of IFNγ on human neurons due to ROS-induced DNA damage, consistent with previous reports of L2/3 neuron loss in an experimental chronic meningeal inflammation model of MS comprising TNF and IFNγ cortical injection in the context of a myelin autoimmune response^45,46^. Fourth, in contrast to the DTA model, gross demyelination did not occur in the AS-IFNγ mice (although myelin sheaths were thinner)^47^. Finally, we found DNA damage and neuronal losses confined to L2/3 that cannot be explained solely by distal demyelination, which would affect other projection neuron populations. Taken together, these lines of evidence suggest that direct neuroinflammatory damage combined with intrinsic vulnerability and loss of trophic support are a primary cause of L2/3 neurodegeneration.

### Implications for MS and new therapies

Cortical atrophy, reflecting degeneration of grey- and white-matter compartments, remains a consistent feature of progressive MS despite effective anti-B and anti-T cell immune therapies^10,11^. Our findings suggest that new therapies to bolster L2/3EN resilience would mitigate cortical neurodegeneration in MS such as antagonizing IFNγ signalling, reducing ROS generation and/or enhancing DNA repair. Indeed, therapies to mitigate oxidative stress (such as lipoic acid and NAC) show promise in terms of atrophy and cognitive outcomes^48,49^. Notably, the *rs10191329*^*AA*^ variant is associated with MS disability progression, chronic inflammation and loss of layer 2 cortical neurons^50,51^, highlighting the need to explore genetic factors that worsen L2/3EN vulnerability.

## Methods

### Human tissue sample collection and compliance

Single-nucleus capture and RNA-seq analysis of age- and sex-matched human control (9) and MS (12) cortices was performed previously^2^. For this study, human post-mortem brain samples with MS grey matter pathology were sourced from the UK Multiple Sclerosis Tissue Bank at Imperial College London. Ethical approval for the use of these samples was granted by the National Research Ethics Committee in the UK (08/MRE09/31). In total, nine snap-frozen brain blocks (four from patients with MS and five from individuals without diagnosed neurological disease) were analysed using immunohistochemistry. For *CUX2* and *ATF4* expression analyses, the 25-year-old sample was obtained from University of California, San Francisco’s Pediatric Neuropathology Research Laboratory and collected in accordance with guidelines established by the Committee on Human Research (UCSF) and approved by the Institutional Review Board. Participant details are provided in Supplementary Table 1.

### Primary rat glial cultures

Fresh Wistar/Han postnatal day 5 rat heads were obtained from Charles River Laboratory (UK) in ice-cold Hibernate-A medium (Thermo Fisher Scientific, A1247501). Whole brains were extracted, homogenized and subjected to centrifugation (3 min, 100*g* at room temperature). The supernatant was removed and the tissue was resuspended in a dissociation solution (34 U ml^−1^ papain (Lorne, LS003126) and 20 μg ml^−1^ DNase type I (Millipore, 10104159001) in Hibernate-A medium) that had been heat-activated at 37 °C and shaken for 20–30 min at 55 rpm and 37 °C. The tissue was centrifuged for 5 min at 200*g*, the supernatant was aspirated and then the tissue was resuspended in a cold neutralizing solution (Hibernate A medium supplemented with 1× B27 (Thermo Fisher Scientific, 17504044) and 2 mM sodium pyruvate (Sigma-Aldrich, S8636)). Tissue was triturated using a serological pipette ten times and, after 1 min rest, the cell suspension (supernatant) was transferred through a 70-μm cell strainer into isotonic Percoll (90%, Sigma-Aldrich, GE17-5445-01). The remaining tissue aggregates were subjected to a further three rounds of trituration and 1 min resting, and the supernatant was transferred into isotonic Percoll. The cell suspensions were brought to a final Percoll concentration of 22.5% by adding DMEM/F12 with HEPES (Thermo Fisher Scientific, 11-039-021) and centrifuged for 20 min at 800*g*. The supernatant was removed, and the brain cell pellet (bottom 3 ml) was resuspended in HBSS without calcium and magnesium (Thermo Fisher Scientific, 14170112), then centrifuged for a further 5 min at 300*g*; the supernatant was next removed and the cells were resuspended in 1 ml of red blood cell lysing buffer (Sigma-Aldrich, R7757). The suspension was incubated for 90–120 s before washing with HBSS without calcium and magnesium. The cells were centrifuged for 5 min at 300*g* before resuspending in 500 μl sorting solution (1× PBS supplemented with 2 mM EDTA (Thermo Fisher Scientific, 15575020), 2 mM sodium pyruvate, 0.5% BSA and 25 μg ml^−1^ insulin (Sigma Aldrich, I9278)). The cells were counted and 2 μl of CD11b/c rat microbeads (Miltenyi Biotec, 130-105-634) was added per 1 million cells and incubated for 15 min at 4 °C with gentle agitation on a tube roller. A further 8 ml of sorting solution was added before the cell/bead mix was centrifuged for 5 min at 300*g*. The supernatant was removed, and cells were resuspended in 2 ml sorting solution. Cells were added to an LS column (Miltenyi Biotec, 130-042-401) fitted to a QuadroMACS Separator (Miltenyi Biotec, 130-091-051) stand that had been pre-wet with sorting solution. The column was washed three times with 2.5 ml cold sorting solution. Microglia were collected from the column by disassociating the column from the stand, adding 2 ml microglial medium (DMEM/F12 supplemented with 60 μg ml^−1^ *N*-acetyl cysteine (Sigma-Aldrich, A9165), 10 μg ml^−1^ insulin, 1 mM sodium pyruvate, 1× SATO (100× SATO: 1.61 mg ml^−1^ putrescine dihydrochloride (Sigma-Aldrich, P5780), 4 µg ml^−1^ sodium selenite (Sigma-Aldrich, S526110), 60 µg ml^−1^ progesterone (Sigma-Aldrich, P8783), 41.25 mg ml^−1^ BSA (Sigma-Aldrich, A4919), 5 mg ml^−1^ apo-transferrin (Sigma-Aldrich, T1147) in DMEM/F12 (Gibco, 11039021)) before using a plunger to gently release the cells. Microglia were counted and seeded at 400,000 cells per well on 12-well plates precoated with 5 μg ml^−1^ poly-d-lysine (Sigma-Aldrich, P6407). To obtain astrocytes, the cell flow-through was further sorted to remove oligodendroglia. Flow-through suspensions were centrifuged for 5 min at 300*g* and the supernatant was aspirated. Cells were resuspended in 500 μl of sorting solution before adding 1.7 μl of mouse IgM anti-A2B5 antibody (about 1:250, Sigma-Aldrich, MAB312, AB-94709) and incubated for 25 min at 4 °C with gentle agitation. The cell suspension was diluted with sorting solution and centrifuged for 5 min at 300*g*. The supernatant was aspirated, and the pellet was resuspended in 160 μl of sorting solution with 40 μl of rat anti-mouse IgM microbeads (Miltenyi Biotec, 130-047-302) and further incubated for 15 min at 4 °C, with gentle agitation at the 5- and 10-min mark. The cells were sorted through the LS column as described above for microglia but the flow-through was collected for further astroglial isolation. The solution was centrifuged for 5 min at 300*g* and then resuspended in astrocyte medium (DMEM/F12 without L-glutamine (Thermo Fisher Scientific, 21331020) supplemented with 1 mM sodium pyruvate, 60 μg ml^−1^ *N*-acetyl cysteine, 1× N2 (Thermo Fisher Scientific, 17502-001), 1× B27 (Gibco, 12587-010), 1% penicillin–streptomycin, 10 μg ml^−1^ insulin and 10 ng ml^−1^ human recombinant HB-EGF (Peprotech, 100-47-100UG)). The cell solution was added to a flask coated with 5 μg ml^−1^ poly-d-lysine and glia were allowed to attach for 1 h at 37 °C. After 1 h, the unattached cells (predominantly containing neurons and endothelia) were washed away, and fresh astrocyte medium was provided. After 24 h, astrocytes were passaged by first washing with PBS minus calcium and magnesium chloride (Sigma-Aldrich, D8537) before replacing with trypsin-EDTA (0.05%; Gibco, 25300054) at 37 °C for 5 min. Astrocyte medium was added and astroglia were collected and centrifuged at 300*g* for 5 min. Astrocytes were resuspended in astrocyte medium and plated at 300,000 cells per well of 12-well plates. Cells were then subjected to rat IFNγ (Thermo Fisher Scientific, 400-20) before collection.

### Demyelination and neuroinflammation models

*ROSA26*-*eGFP-DTA* mice^52^ were bred to the *PLP/creER*^*T*^ transgenic mice^53^ to generate the *PLP/creER*^*T*^;*ROSA26*-*eGFP-DTA* (DTA) mice, as previously described^17,18^. All mice were on the C57BL/6J background. *PLP/creER*^*T*^;*ROSA26*-*eGFP-DTA* (DTA) mice and *ROSA26*-*eGFP-DTA* littermates (control; aged 5–7 weeks) were injected intraperitoneally with 0.8 mg of 4-hydroxytamoxifen (Hello Bio, HB6040) per day, either for 4 consecutive days (males) or 3 consecutive days (females). Brain tissue was collected from the DTA and control animals at the indicated timepoints after tamoxifen injection. Equal numbers of male and female mice were used for experiments, which were conducted in compliance with Northwestern University’s Animal Care and Use Committee (IACUC) guidelines.

*Myrf*^*fl/fl*^ were crossed to *Sox10*^*creERT*^ mice to generate *Myrf*-cKO mice as previously described^20^. All of the mice were on a mixed C57BL/6N and C57BL/6J background and *creERT-*negative littermates served as non-demyelinated controls. Eight-week-old mice were injected intraperitoneally with 100 mg per kg tamoxifen (T5648, Sigma-Aldrich) dissolved in corn oil (C8267, Sigma-Aldrich) for 5 days. These experiments were conducted in compliance with the Institutional Animal Care and Use Committee of OHSU.

### Ectopic IFNγ expression model

*Gfap-tTA* mice on the C57BL/6J background were crossed with *TRE-Ifng* mice on the C57BL/6J background to generate *Gfap-tTA;TRE-Ifng* double-transgenic mice, as described previously^54,55^. To suppress IFNγ transcription, doxycycline (200 ppm) was administered in the diet (Envigo) from conception. Mice were maintained on doxycycline throughout development, and doxycycline repression was lifted at 8 weeks of age. Brain tissue was collected from both double-transgenic and single-transgenic control mice at 12 months after doxycycline withdrawal. These experiments were conducted in compliance with Northwestern University’s Animal Care and Use Committee (IACUC) guidelines.

### KO and cKO lines

*Cux2*^*cre*^^56,57^, *Cux2*^*creER*^^57^, *Atf4*^*fl*^^58^ and WT mice were handled according to guidelines set by the University of California, San Francisco and housed within a barrier facility on a 12 h–12 h light–dark cycle. Mice were housed with up to four other same-sex cage mates in standard rodent cages and provided food and water ad libitum. Both male and female mice were used for all experiments. All animal protocols and procedures were approved by UCSF’s Institutional Animal Care and Use Committee (IACUC).

### Neonatal hypoxia and acute neuroinflammation model

Neonatal mice, along with their parents, were exposed to chronic hypoxia by receiving 10% fractional inspired oxygen from P3 to P14. At P7, LPS (Sigma-Aldrich, L2630, 93572-42-0) was administered through intraperitoneal injection at a dose of 2 mg per kg. After P14, both pups and parents were returned to normoxic conditions until brain tissue was collected at P28. Mice were monitored daily. The oxygen concentration was continuously monitored and maintained using an oxygen modulator (Biospherix, ProOx110). This procedure was approved by UCSF’s Institutional Animal Care and Use Committee (IACUC).

### Acute demyelination with cuprizone

Tamoxifen (T-5648, Sigma-Aldrich, 10540-29-1) was dissolved in a solution of 90% corn oil and 10% ethanol and administered intraperitoneally to *Cux2*^*creER*^*Atf4*^*fl*^ mice starting at 8 weeks of age. Each mouse received 2 mg of tamoxifen per day for 5 consecutive days, followed by a 2 week rest period.

Next, the same mice were fed a diet containing 0.3% (w/w) cuprizone (Sigma-Aldrich, C9012, 370-81-0) mixed into standard chow. Cuprizone was administered continuously for 6 weeks to induce robust demyelination. Mice were monitored daily and provided with fresh cuprizone-containing food until brain tissue was collected at the end of the 6-week period. This procedure was approved by UCSF’s Institutional Animal Care and Use Committee (IACUC).

### Collection of tissue for histology

For histology, mice were transcardial perfused with 4% paraformaldehyde (PFA), and brain tissue was collected. The brains were post-fixed overnight in 4% PFA and washed with PBS before being embedded in OCT. Then, 16-μm sections were obtained on Superfrost Plus slides using a Leica Cryostat. The sections were stored at below −70 °C.

### Collection of tissue for snRNA-seq

For snRNA-seq, cortices were collected in ice-cold DEPC-PBS under RNase-free conditions, frozen with dry ice and stored at −80 °C until sequencing was performed.

For snRNA-seq on DTA mice, 1 mm coronal sections were collected using a stainless-steel brain matrix. From these sections, 0.75 mm punches were collected from the corpus callosum and cortex, which were frozen and shipped to UCLA, where the sequencing was performed.

### snRNA-seq

For snRNA-seq, mRNAs from isolated nuclei were barcoded using the 10x Genomics 3′ Gene Expression v3.1 kit. Libraries were sequenced on the NovaSeq 6000 system using an S4 flow cell. Raw data were processed with CellRanger (v.7.0.1) using the mm10-2020-A mouse genome reference for embryonic samples or CellRanger (v.8.0.1) with GRCm39-2024-A mouse reference for postnatal samples. Ambient RNA contamination was removed using CellBender (embryonic, v.0.1.0; postnatal, v.0.3.0).

### Cell type annotation

Cell type annotation was performed using a combination of clustering and reference-based approaches. We used the Deep Embedding for Single-cell Clustering (DESC, v.2.1.1)^59^ package for dimension deduction, batch normalization and clustering, using the top 2,048 high DEGs (scanpy^60^, v.1.8.1) and a three-layer encoder network (1,024, 256, 32) for feature extraction. Clusters were annotated using Pegasus (v.1.8.1) for automated cell type suggestions, with further validation against the Allen Brain Atlas and the Linnarsson Atlas for cortical regions^61,62^. For the integrated Popko dataset, a hierarchical annotation strategy was applied, sequentially removing non-neuron cells, non-cortex neurons and inhibitory neurons, with manual marker gene verification at each step.

### DEG analyses

DEGs for *Cux2*^*cre*^*Atf4*^*fl*^ mice were determined in Omics playground^63^ (v.2.8.19) by performing *t*-tests (standard, Welch) and limma (no trend, trend, voom), edgeR (QLF, LRT) and DESeq2 (Wald, LRT) tests and taking the highest *q* value for tests with cutoffs of a false-discovery rate (FDR) of 0.05 and a log_2_-transformed FC of 0.1.

DEGs for DTA mice were determined instead in Python using Scanpy’s rank_genes_groups function and using Wilcoxon rank-sum tests to determine statistical significance.

Volcano plots were generated using the Hiplot (ORG) (https://hiplot.org) Volcano plot APP (v.0.1.0) using a Creative Commons Attribution 4.0 International Licence to visualize DEGs with highlighted genes. Moreover, DEG distribution box plots were created by randomly sampling 1,000 cells from each cluster, repeated 100 times. The filtered number of DEGs (*P* < 0.05 and log_2_[FC] > 0.1) in each cluster was recorded as a .csv file and then visualized using ggplot2 v.3.5.1. Minor visualization modifications were made to plots in Adobe Illustrator such as removing unwanted lines and text formatting.

### Trajectory pseudotime and real-time analysis

Pseudotime analysis was based on previously established trajectories from our previous sequencing study^2^. Gene expression dynamics over pseudotime and real time were analysed by calculating the mean gene expression or gene list scores at each timepoint, followed by polynomial fitting using numpy. For pseudotime analysis, four timepoints were estimated based on the region where the cells are from (healthy controls, normal appearing white matter, acute lesion and chronic lesion region), while for real-time analysis, the timepoints are based on the age of the animals when collected. Gene list scores were calculated using Scanpy’s score_genes or Seurat’s AddModuleScore function, with 500 random genes selected as negative controls to establish baseline scores.

Interactive cell annotation and analyses were performed using Cellxgene-VIP (v.3.0) and Omics playground.

### GO analysis

DNA repair GO analysis (Supplementary Table 3) was performed in g.profiler (v.4.2.8)^64^ (https://biit.cs.ut.ee/gprofiler/gost) with statistical enrichment calculated using the g:SCS test with experiment-wide threshold of *α* = 0.05.

GO analysis on DEGs was conducted and visualized using the ClusterProfiler^65^ (v.4.14.6) package in R. DEGs identified by Scanpy’s rank_gene_groups function (*P* < 0.05, log_2_[FC] > 0.2) were used as input. Enrichment significance was assessed using thresholds of *P* < 0.05 and *q* < 0.05, and redundant GO terms were reduced using the simplify() function.

### DDR gene list

We compiled the list of 698 genes considered as DDR genes by combining GOterms GO:0006281DNArepair and HALLMARK DNA_REPAIR (M5898), and consolidating with previously curated human DNA repair genes by R. Wood and M. Lowery (https://www.mdanderson.org/documents/Labs/Wood-Laboratory/human-dna-repair-genes.html)^66–68^.

### Quantitative PCR

RNA was isolated from cells in culture by first washing with 1× PBS and then replacing PBS with TRI reagent (Cambridge Bioscience, R2051) for 5–10 min at room temperature. RNA was isolated using the Direct-zol RNA miniprep kit (Cambridge Bioscience, R2051) according to the manufacturer’s instructions. cDNA was generated using the Zymoscript RT Premix kit (Cambridge Bioscience, R3012). cDNA (approximately 20–200 ng diluted in H_2_O) was amplified in a mastermix containing 0.1 μM forward and reverse primers (Supplementary Table 12) and PowerUp SYBR Green Master Mix (1×, Applied Biosystems, Thermo Fisher Scientific, A25777). Quantitative comparative PCR was performed on the Applied biosciences Quantstudio 12K Flex Real-time PCR system (Thermo Fisher Scientific, 4470661) using the default SYBR comparative CT program.

### Neurogenin-2 knock-in iPS cells

Before nucleofection, the ribonucleoprotein complex was assembled and incubated at room temperature. Twenty μg of Cas9 (Integrated DNA Technologies, 1081061) was complexed with 150 pmol of the synthetic gRNA targeting the AAVS1 locus in nuclease-free duplex buffer (Integrated DNA Technologies, 11-01-03-01). After 30 min incubation, 2 μg of the donor plasmid pUCM-AAVS1-TO-hNGN2-T2A-Zeo was added and mixed with 10^6^ cells (iPS cells, CAMi014-A (corrected line))^69^ that were then nucleofected in 100 μl cuvettes using the Amaxa 4D-Nucleofector (Lonza) and the P3 Primary Cell 4D-Nucleofector X Kit (Lonza, V4XP-3024) using the CA-137 program. Cells were then plated with 4 μM Cas9 Electroporation Enhancer (Integrated DNA Technologies, 1075916) and 10 μM of ROCKi. The medium was refreshed after 24 h.

Then, 3 days after nucleofection, the cells were pooled and assessed for mCherry expression by flow cytometry using the CytoFLEX (Beckman Coulter). By plotting SSC-H-Lin versus FSC-H-Lin live cells, the population was revealed (centre) and gated. The single-cell population was isolated by plotting the live cell subpopulation on an FSC-H-Lin versus FSC-ALin. Single cells were analysed for mCherry expression by plotting FSC-H-Lin versus PE-A-Log; mCherry positive population was gated to the right of the 10^4^ mark on the PE-A axis. Flow cytometry data were analysed using FlowJo v.10.7.1 (Becton Dickinson). Simultaneously, cells were observed for mCherry expression on the EVOS fluorescence microscope. Recombinant cells were selected with 1 μg ml^−1^ puromycin for 5 days.

After puromycin selection, clones were isolated by plating 10^3^ filtered cells with a 30 μm mesh in a 10 cm dish and picked when colonies were visible by eye. Colonies were expanded and selected based on the ability to differentiate into *NGN2-*iNs.

### Cell lines and culture

SHSY5Y neuroblastoma cells were obtained from ATCC (CRL-2266) and maintained in DMEM/F-12 (Sigma-Aldrich, D8437) supplemented with 10% FBS and penicillin–streptomycin (LifeTech/Gibco, 15140-122; neuroblastoma medium) and cryofrozen with the addition of 10% DMSO (Sigma-Aldrich, D2438). Cells were passaged by washing once with PBS minus calcium chloride and magnesium chloride (Sigma-Aldrich, D8537) and replacing with trypsin-EDTA (0.05%; Gibco, 25300054) at 37 °C for 2 min. Cells were collected in neuroblastoma medium and pelleted by centrifugation at 300*g* for 5 min. Cell pellets were resuspended in neuroblastoma medium.

*NGN2-*iPS cells were maintained in Stemflex medium (Thermo Fisher Scientific, A3349401) on six-well plates coated with Corning Matrigel growth factor reduced basement membrane matrix (Corning, Scientific Laboratories Supplies, 356230). Cells were passaged by washing once with PBS minus calcium chloride and magnesium chloride (Sigma-Aldrich, D8537) and incubating in 0.5 mM EDTA in 1× PBS (Thermo Fisher Scientific, 15575020) for 5 min at 37 °C. Cells were collected in Stemflex and pelleted by centrifugation at 300*g* for 5 min. Cell pellets were resuspended in Stemflex supplemented with 10 μM ROCK inhibitor Y-27632 (Abcam, ab120129) and either plated for further growth or cryofrozen by addition of 10% DMSO.

hTERT RPE-1 cells were obtained from ATCC (CRL-4000) and maintained in RPE-1 medium: DMEM/F12 high glucose (Thermo Fisher Scientific, 31053028) supplemented with 10% FBS, 1× MEM-NEAA (Life Technologies, 11140035) and 1× GlutaMax (Gibco, 31331028). Cells were passaged as described for SHSY5Y.

All of the cell lines tested negative for routine *Mycoplasma* testing by PCR and rapid test. No cell lines were authenticated.

### Induced neuron generation and culture

*NGN2-*iPS cells were induced to become neurons as follows: *NGN2-*iPS cells were dissociated into single cells using Accutase (in place of EDTA, Life Technologies, A1110501) at 60–80% confluence and 250,000 cells per well were plated in six-well culture dishes coated with Corning Matrigel growth-factor-reduced basement membrane matrix. On day 1 and 2, the medium was replaced with fresh D1-2 differentiation medium (DMEM/F12, GlutaMax (Gibco, 10565-018) supplemented with 1× MEM-NEAA (Life Technologies, 11140035), 1% penicillin–streptomycin, 1× N2 (Thermo Fisher Scientific, 17502-048), 1 μg ml^−1^ doxycycline (Sigma-Aldrich, D9891, 24390-14-5) and 55 μM 2-mercaptoethanol (Gibco, 21985023)). On day 3, the medium was replaced with D3+ differentiation medium (Neurobasal (Gibco, 21103-049), supplemented with 1× B27 (Gibco, 12587-010), 1% penicillin–streptomycin, 1× GlutaMax, 55 μM 2-mercaptoethanol, 1 μg ml^−1^ doxycycline, 10 ng ml^−1^ NT3 (Peprotech, 450-03) and 10 ng ml^−1^ BDNF (Peprotech, 450-02)). On day 4, 96-well plates were coated with poly-l-lysine (0.1 mg ml^−1^, Sigma-Aldrich, P1524) for 1 h at 37 °C. Induced neuron cultures were washed once with PBS minus calcium chloride and magnesium chloride and Accutase was added for 5 min at 37 °C. Cells were collected in 1× PBS and pelleted by centrifugation for 5 min at 300*g*. Cells were resuspended in D3+ medium supplemented with 10 μM ROCK inhibitor Y-27632. Cells were plated at 100,000 cells per well of a 96-well or 24-well plate. On day 5 and 6, medium was refreshed with D3+ differentiation medium. From day 8, cells were maintained by replacing half of the medium with D3+ differentiation medium minus doxycycline every other day.

### Plasmid construction, lentivirus preparation and infection

Lentivirus donor DNA plasmids were ordered from Vectorbuilder and were as follows: pLV[Exp]-Puro-CBh>hCUX2(NM_015267.4)/GST/T2A/eGFP (CUX2-OE), pLV[Exp]-Puro-CBh>hATF4(NM_001675.4)*/Flag/T2A/mCherry (ATF4-OE) or pLV[Exp]-Puro-CBh>eGFP (GFP-OE). The specific constructs used for hTERT RPE-1 cell overexpression were as follows: pLV[Exp]-Puro-CBh>hATF4(NM_001675.4]/3×GS/Halo, pLV[Exp]-Puro-CBh>hCUX2(NM_015267.4)*/3×GS/Halo and pLV[Exp]-Puro-CBh>hRPA3(NM_002947.5)/HA/T2A/eGFP.

HEK293T cells were sourced from ATCC (CRL-3216) and maintained as described for the neuroblastoma cell line but were cultured without antibiotics. To generate lentivirus, HEK293T cells were grown in 15 cm dishes and split 1:1 at 70% confluence 1 day before transfection. Then, 4.5 μg of psPAX2 (Addgene, 12260) and 4.5 μg of vSVG (Addgene, 14888) were combined with 4.5 μg of donor DNA in 2.25 ml Opti-MEM reduced serum medium. Next, 54 μl of Lipofectamine 2000 Transfection Reagent (Thermo Fisher Scientific, 11668030) was combined with 2.25 ml Opti-MEM reduced serum and then mixed with the DNA/Opti-MEM solution and incubated at room temperature for 20 min. DNA–lipid complexes were added to each 15 cm plate and incubated for 15 h in the incubator. The medium was changed to target cell medium (for example, neuroblastoma medium) and the supernatant was collected after a further 24 h, refreshed and then collected again after another 24 h. The collected supernatants were spun at 500*g* for 10 min to remove cell debris and filtered through 0.45 µm filter before being aliquoted and stored colder than −70 °C until use.

Lentiviral transduction was performed by combining lentivirus-containing supernatants 1:1 with fresh complete medium (neuroblastoma or D3+) and applying to neuroblastoma or *NGN2-*iNs in 24-well or 6-well plates. Spinoculation was performed at 25–35 °C by centrifugation at 1,000*g* for 30 min to 1 h. The medium was changed the next day for *NGN2-*iNs or after 48 h for neuroblastoma. Lentiviral-transduced cell lines were established by selecting with puromycin (10 µg ml^−1^; Thermo Fisher Scientific, J67236.XF) from day 3 after transduction.

### Comet assays

Neuroblastoma cells were plated at 500,000 cells per well in six-well plates. The next day, cells were exposed to 200 μM TBHP (Sigma-Aldrich, 458139, 75-91-2) or left untreated for 1 h. Treated cells were recovered for 0–4 h before collecting cells in 1× PBS by scraping. Cell suspensions were then combined 1:4 with low-melting-point Comet agarose (R&D systems, 4250-050-02) and 75 μl was applied to a single well of a Comet slide (R&D systems, 4250-200-03). The slides were set for 15 min at 4 °C, before being subjected to lysis solution (R&D systems, 4250-050-01) for 1 h at 4 °C. Alkaline unwinding was performed at room temperature for 20 min by submerging the slides in 300 mM NaOH, 1 mM EDTA, in distilled H_2_O. The slides were transferred to an electrophoresis chamber submerged in alkaline unwinding solution and run at 1 V cm^−1^ for 20 min (where cm = distance between electrodes). After electrophoresis, slides were submerged in 70% (w/v) ethanol in distilled H_2_O for 5 min and dried overnight at room temperature. Cells were stained by applying SYBR green stain diluted in 1 mM EDTA and 10 mM Tris-HCl (Thermo Fisher Scientific, 10573145). Images were collected on the EVOS FL upright microscope.

### MTT viability assay

Neuroblastoma cells were plated at 40,000–50,000 cells per well in 96-well plates. *NGN2-*iNs were plated at 100,000 cells per well on day 4. On day 5 (neuroblastoma) or day 19 (*NGN2-*iNs), a mastermix containing DNA-damaging agents (TBHP; Carboplatin, Sigma-Aldrich, C2538, 41575-94-4; 6-thioguanine, Sigma-Aldrich, A4882, 154-42-7; thapsigargin, Abcam, AB120286, 67526-95-8; topotecan, Apex Bio, B2296, 119413-54-6; colchicine, Sigma-Aldrich, C975, 64-86-8; etoposide, Sigma-Aldrich, 341205, 33419-42-0) diluted in fresh medium was made, and 100 μl was delivered to each well. Then, 24 h later, MTT reagent (Abcam, ab211091) was mixed 1:1 in DMEM (Thermo Fisher Scientific, 11-039-021) and added to cells for 45 min to 1 h at 37 °C and 5% CO_2_. MTT solution was removed and replaced with pure methanol (100 μl per well of a 96-well plate). Colorimetric cell viability was measured on a Spectrostar nano (BMG Labtech) plate reader using Spectrostar nano firmware (v.1.11) and software (v.2.12) at 570 nm and 690 nm.

### CellROX probe assay

Primary rat astrocytes, microglia or human *NGN2-*iNs were pretreated with IFNγ (Thermo Fisher Scientific, human: PHC4031; rat: 400-20), and supplemented with 10 μM NAC (Sigma-Aldrich, A9165-5G) or 1 μM Mito-TEMPO (Sigma-Aldrich, SML0737). CellROX reagent (5 μM; Invitrogen, C10448) was added to the medium and cells were returned to the 37 °C incubator for 30 min. Images were collected on an EVOS FL upright microscope and analysed for fluorescence using a custom CellProfiler pipeline (v.4.2.8; available at GitHub: https://github.com/RowitchLab/Code_for_Cux2_Atf4_paper).

### Luciferase assay

The human *RPA3* promoter (1,500 bp upstream to 100 bp downstream of the transcriptional start site) was cloned into the pGL4.10[luc2] vector (Promega, E6651) using Kpn1 digestion and Infusion cloning (Takara, 638945) with specific primers (Supplementary Table 12). The following vectors derived from Vectorbuilder were used to express GFP and CUX2: pRP-CBH-ORFstuff-eGFP and pRP-CBH-hCUX2(NM_015267.4)-eGFP.

In total, 100,000 HEK293T cells were plated in 96-well dishes and transfected the next day with pGL4.10[luc2] or pGL4.10[Rpa3-luc2] (325 ng per well), overexpression vectors (9.55 fmol) and pGL4.75[hRluc/CMV] (5 ng per well) vectors using Lipofectamine 2000 Transfection Reagent. Then, 24 h later, a dual-luciferase reporter assay (Promega, E1910) was performed according to the manufacturer’s instructions and firefly luciferase and *Renilla* luciferase activity was measured on the Promega Glomax luminometer. Firefly luciferase readings were normalized to *Renilla* luciferase as an internal transfection control.

### NHEJ reporter assay

Non-blunt NHEJ reporter plasmids were a gift from E. Rajendra (Artios Pharma) and prepared by I-SceI digestion and purification as described previously^70^.

A total of 200,000 hTERT RPE-1 cells was transfected with siRNA (Supplementary Table 12) using Lipofectamine RNAiMAX (Thermo Fisher Scientific, 13778100) at passage according to the manufacturer’s instructions and plated immediately in six-well dishes. After 72 h, cells were passaged by washing once with PBS minus calcium chloride and magnesium chloride and replacing with trypsin-EDTA (0.05%; Gibco, 25300054) at 37 °C for 2 min. Cells were collected in RPE-1 medium and pelleted by centrifugation at 300*g* for 5 min. Cell pellets were resuspended in RPE1 medium and counted. Meanwhile, non-blunt NHEJ substrate (0.5 μg DNA per 1 × 10^6^ cells) was combined with the pGL4.50[luc2/CMV/Hygro] Vector (Promega, E1310; 0.66 μg DNA per 1 × 10^6^ cells) in JetPRIME buffer (Sartorius, 201000003). Diluted DNA was then complexed with JetPRIME reagent (2.34 μl reagent per 1 × 10^6^ cells, Sartorius, 101000046) and incubated for 10 min at room temperature. Cells were then combined with DNA transfection complexes and plated at 27,000 cells per well in 96-well plates. Then, 24 h later, cells were washed with PBS, lysed with 20 μl of 1× passive lysis buffer (Promega E1941) for 15 min at room temperature. The Nano-Glo Dual-Luciferase Reporter Assay (Promega, N1610) was performed according to the manufacturer’s instructions. Firefly and NanoLuc luciferase were detected using the Promega Glomax luminometer. NanoLuc measurements (NHEJ repaired substrate) were normalized to the Firefly measurement to account for variability in transfection efficiency. Readings from 10 ×100,000 plated cells were averaged per trial.

### Immunohistochemistry analysis of rodent brain tissue

Cryosections were removed from the freezer and air dried for 15 min at room temperature. Slides were washed for 10 min in 1× PBS before antigen retrieval was performed either (1) in a steam pot for 5 min immersed in sodium citrate buffer (10 mM sodium citrate, 0.05% Tween-20, pH 6.0); or (2) in an 80 °C water bath immersed in 1× citrate buffer (Sigma-Aldrich, C9999) for 30–60 min. The slides were washed in 1× PBS for 10 min twice, before blocking solution was added for 1 h (10% normal donkey or goat serum in 0.1% Triton X-100/1× PBS). Primary antibodies (Supplementary Table 11) were added to blocking solution and incubated on slides overnight at 4 °C or room temperature. The slides were washed in 1× PBS for 10 min thrice. Secondary antibodies (Supplementary Table 11) were diluted in 0.1% Triton X-100/1× PBS and incubated for 1 h on slides. The slides were washed three times in 1× PBS for 10 min each, before DAPI (0.25 μg ml^−1^, Sigma-Aldrich) or Hoechst (1:1000, Life Technologies, H3570) was added for 20 min at room temperature. Slides were coverslipped in ProLong Gold Antifade Mountant (P36930, Thermo Fisher Scientific).

### In situ hybridization on rodent brain tissue

For in situ hybridization staining, tissue sections were incubated overnight at 65 °C with a diluted, denatured DIG-labelled antisense mouse *Cux2* probe. This was followed by three post-hybridization washes at 65 °C and two additional washes at room temperature. Next, the sections were incubated overnight at 4 °C with an anti-digoxigenin-AP Fab fragments antibody (1:1,500, 11093274910, Sigma-Aldrich). The next day, targeted mRNA-expressing cells were visualized as a dark purple deposition using the NBT/BCIP–alkaline phosphatase reaction (11681451001, Sigma-Aldrich).

### Immunohistochemistry staining of human post-mortem brain tissue

To classify demyelination in post-mortem human brain tissue, the sections were stained using immunohistochemistry with diaminobenzidine (DAB, SK-4105, Vector Laboratories) for the detection of myelin oligodendrocyte glycoprotein (MOG). Tissue sections were initially fixed in 100% methanol at −80 °C for 5 min at room temperature, followed by a 5 min PBS wash at room temperature. A hydrophobic barrier was then created around the sections using a PAP pen (VEC-H-400, Biozol), after which they were blocked with 10% goat serum (16210064, Thermo Fisher Scientific) diluted in 0.01% PBS/Triton X-100 for 30 min at room temperature. After the blocking step, sections were incubated overnight at 4 °C with the primary MOG antibody (MAB5680, Merck Millipore). The next day, the sections were rinsed with PBS and then incubated for 2 h at room temperature with a biotinylated secondary IgG antibody. After 2 additional PBS washes, the sections were treated with an avidin-biotin complex for 1 h at room temperature. The DAB substrate was applied until a colour change was visible under the microscope. Subsequently, the sections were dehydrated using a gradient series (50%, 70%, 96%, 100%) of ethanol and cleared with two 10-min incubations in 100% xylene. Finally, the sections were mounted using Eukitt (Orsatec).

Human fresh frozen brains sections were fixed in 4% PFA for 10 min at room temperature. After fixation, the sections were rinsed three times with 1× PBS to remove any residual fixative. A hydrophobic barrier was created around the tissue sections using a PAP pen. The tissue sections were simultaneously blocked and permeabilized by incubating them in 5% goat serum diluted in 0.1% PBS-Triton X-100 for 1 h at room temperature. After blocking, the sections were briefly washed with 0.05% PBS/Tween-20 for 2 min. The primary antibodies (Supplementary Table 11) were incubated overnight at 4 °C in 0.05% PBS/Tween-20. The next day, the slides were washed three times with 1× PBS for 2 min each, followed by a secondary antibody (Supplementary Table 11) incubation for 1 h at room temperature. After secondary antibody staining, the slides were washed again with 1× PBS for 2 min. Finally, the sections were mounted using Fluoromount mounting medium containing DAPI (15596276, Invitrogen).

### Immunocytochemistry

Cells were cultured in glass bottom 24-well plates (P24-1.5H-N, Cellvis), washed twice with 1× PBS and then fixed in 4% PFA for 15 min at room temperature. Fixed cells were washed in 1× PBS for 10 min thrice. Blocking solution (10% normal donkey serum in 0.1% Triton X-100/1× PBS) was added for 30 min at room temperature before primary antibodies (Supplementary Table 11) were added to blocking solution and incubated with fixed cells for 3 h at room temperature. Antibody solution was removed and the wells washed in 1× PBS for 10 min thrice. Secondary antibodies (Supplementary Table 11) were diluted in 0.1% Triton X-100/1× PBS and incubated for 1 h. Antibody solution was removed and the wells washed in 1× PBS for 10 min thrice. Wells were incubated with Hoechst (1:1,000, X) for 5 min at room temperature followed by three more washes in 1× PBS for 5 min each.

### smFISH

Single-molecule fluorescence in situ hybridization (smFISH; RNAscope) analysis of rodent tissue sections was performed using the RNAscope LS Multiplex Assay Kit (Advanced Cell Diagnostics, ACD) as previously described^2^. z-probes with one of four distinct tail configurations (C1–C4) were obtained from ACD (Supplementary Table 12). Slides with cryosections were thawed at room temperature for 15 min before incubating at 65 °C for 45 min in an oven. Slides were immersed in 4% PFA at 4 °C for 15 min and then washed twice with 1× PBS. Slides were then transferred through an ethanol solution series: 5 min each at 50%, 70%, 100% and 100% ethanol in water. Coverplates (S21.4611, Leica) were added and slides were inserted into the Leica BOND RX. The slides were then exposed to ER2 (AR9640, Leica) at 95 °C for 5 min and subsequently, ACD Protease III at 42 °C for 20 min. ACD hydrogen peroxide was added for 10 min at room temperature to inactivate endogenous peroxidases and ACD protease III. Probe master mixes were produced by diluting z-probes in ACD Blank Probe Diluent master (300048, Biotechne). Probe mixes were hybridized to sections for 2 h at 42 °C. Sequential incubations in AMP1, AMP2 and AMP3 reagents for 30 min each at 42 °C allowed branched DNA tree development. The samples were then incubated with their corresponding HRP reagent for 1 min at 42 °C. TSA Opal 520, TSA Opal 570, TSA Opal 650 and TSA Opal 690 (Akoya Biosciences, FP1487001KT, FP1488001KT, FP1496001KT, FP1497001KT) were incubated at 1:500 concentration in TSA buffer for 30 min at 42 °C, followed by blocking with HRP blocking reagent for 15 min at 42 °C. For probes using ATTO 425 dye, the probe was incubated with TSA-biotin (Akoya, 1:500) for 30 min at room temperature, followed by streptavidin conjugated ATTO 425 (Sigma-Aldrich, 1:400) for 30 min. The samples were incubated in DAPI (0.25 μg ml^−1^, Sigma-Aldrich) for 20 min at room temperature. The slides were removed from the Leica BND RX and coverslipped with ProLong Gold Antifade Mountant (P36930, Thermo Fisher Scientific).

To identify human neurotypical CUX2 and ATF4 co-expression, the slide preparation protocol was modified as follows: the slides were first baked for 20 min at 60 °C before fixing with 4% PFA for 10 min. After a PBS wash of 5 min, slides were incubated in hydrogen peroxide and protease reagent (322381, ACD Bio) for 10 min, then subjected to antigen retrieval in Target retrieval buffer (322000, ACD Bio) at 95 °C for 5 min. The sections were then washed in distilled water and dehydrated in 100% ethanol. 3-plex RNAscope was performed using tyramide signal amplification with Opal 520, 570 and 690 dyes to label the probes.

### Immunoblotting

Cells were collected in RIPA buffer (Sigma-Aldrich, R0278) supplemented with 1× protease inhibitor cocktail (Thermo Fisher Scientific, 78425), incubated on ice for 30 min with intermittent vortexing. Lysates were frozen at −80 °C until processing. The samples were centrifuged at 13,000*g* for 10 min at 4 °C and the supernatant was collected and suspended in 1× Bolt LDS sample buffer (Thermo Fisher Scientific, B0007) and 1× Bolt sample reducing agent (Thermo Fisher Scientific, B0009) before being boiled at 70 °C for 10 min. Proteins were separated by gel electrophoresis within 4–12% Bolt Bis-Tris gels (Thermo Fisher Scientific, NW04125BOX) before being transferred to PDVF membranes at a constant 12 V at 4 °C overnight. Membranes were blocked in 5% non-fat milk diluted in 1× Tris-buffered saline with 1% Tween-20 (TBST) for 1 h, washed twice and incubated with primary antibodies (1:1,000; Supplementary Table 11) diluted in 1× TBST overnight at 4 °C. After three 10 min washes in 1× TBST, membranes were incubated in HRP conjugated antibodies (Supplementary Table 11) diluted in 1× TBST for 1 h at room temperature. After three more washes in 1× TBST, membranes were reacted with SuperSignal West Pico PLUS Chemiluminescent Substrate (Thermo Fisher Scientific, 34580) and visualized on a ChemiDoc system (Bio-Rad). Phosphorylated ATM bands were stripped using Restore Western Blot Stripping buffer (Thermo Fisher Scientific, 21059) before membranes were probed for total ATM. Bands were identified and quantified in ImageLab software (v.6.1, Bio-Rad).

### Imaging

Standard imaging of immunohistochemistry or in situ hybridization was performed on the ZEISS Axio Imager 2 using Zen Blue Pro software (v.2.6). Confocal images were acquired on an Operetta CLS High Content Analysis System (Perkin Elmer) using the spinning-disc confocal mode with a sCMOS camera and a ×40/1.1 NA apochromatic water dispensing objective with eight separate LED light sources and acquired using Harmony software (v.4.9). Each *z*-stack consisted of exactly 21 planes with a 1 µm step size and tiled images were taken with 3% overlap. Images were stitched using a Perkin-Elmer tool (Acapella v.5.3.1).

For DNA damage immunohistochemistry and any immunocytochemistry, images were acquired on a Leica SP5 confocal system fitted with DMI8 inverted microscope stand and a 40×/1.3 NA Plan Apo objective using a 405 nm diode laser, 458, 488 and 514 nm lines of an argon laser, plus 561 nm HeNe and 633 nm HeNe lasers and detected with 2 PMT and 2 Leica HyD detectors. Images were stitched and projected using Leica Application Suite X (v3.5.7.23225) software. For imaging of post-mortem human RNAscope, a Leica Stellaris SP5 confocal system was used. Leica Stellaris confocal system was fitted with DMI8 inverted microscope with a HC PL APO CS2 ×40/1.30 NA oil objective, Diode 405, Diode 638, OPSL488 and OPSL 561 nm lasers and detected with Trans PMT and Leica HyD detectors. Images were stitched and projected on Leica LASX software. Stained sections from post-mortem human brain samples were visualized using a Leica DM6 B Thunder microscope equipped with a Leica K5C camera. Images were collected at ×40 magnification for high-resolution visualization of the target probes. To capture the entire signal within the tissue sections, images were acquired as *z*-stacks based on Nyquist sampling criteria.

### Layer thickness and cortical cell counts

Regions of interest were analysed from 1–3 consecutive 16 μm sections over the primary somatosensory barrel field and averaged. Layers were identified by cell morphology changes based on both Hoechst or DAPI in combination with NeuN staining. Layer thickness was measured in Fiji v.2.14.0/1.54f (ImageJ.net) and cell counts were manually taken using the ImageJ Cell Counter plugin and averaged. The researcher was blinded to the experimental conditions.

### Quantification of DNA damage marks

Regions of interest from the mouse cortical L2/3 (1.2 × 10^5^ μm from 1–3 consecutive 16 μm sections) and the human cortical L2/3 (4.9 × 10^5^ μm) were analysed for 53BP1^+^ and γH2AX^+^ foci within NeuN stained cells. The ImageJ Cell Counter plugin (on Fiji v.2.14.0/1.54 f) was used to track cell characteristics and the number of γH2AX^+^ foci was manually recorded per cell. The researcher was blinded to the experimental conditions, with the exception that two human neurotypical controls were added after the initial blinded analysis because one of the blinded neurotypical samples had to be removed as the brain showed Alzheimer’s pathology and cortical atrophy.

### Study design and statistical analysis

Sample size was determined based on the likelihood of phenotypic effects observed in previous studies from the same human samples and animal models used^2,17,18,20,52–55,58,71^, while also considering the availability of sex and age matched samples. All sample sizes are individually reported in figure legends. All data were produced from independent repeated experiments using biological replicates as stated in the figure legends and methods. All samples were randomized with the exception that equal male and female mice were allocated to experimental groups amongst littermates. Experimenters were not fully blinded to group allocation during data collection, but sample IDs without group information were used to deter bias and all experimental parameters were kept consistent across groups, so there are no differences between replicates/groups. Experimenters were blinded to the experimental parameters during data analyses unless the analyses involved automated quantification whereby experimental parameters were applied consistently across all samples. Two human neurotypical controls were analysed after the initial blinded analysis as one blinded sample was removed due to conflicting Alzheimer’s pathology. All seven remaining samples were quantified with the researcher blinded to the experimental condition. Subjective measurements were not used in this study.

Statistical tests on RNA-seq data were determined using Wilcoxon rank-sum tests performed in R software unless otherwise stated in the figure legends. Statistical analysis on cell culture and histological data were performed in Prism software (v.10.4.1). The samples were first tested for normality and then statistical comparisons were made using either the parametric or nonparametric counterparts as reported in the figure legends. Images were pseudocoloured in Adobe Photoshop (v.26.4.0) and the brightness and contrast were adjusted consistently across samples. The figures were finalized in Adobe Illustrator (v.30.1).

### Materials availability

All unique materials generated in this study can be obtained from the lead contact upon completion of a materials transfer agreement.

### Reporting summary

Further information on research design is available in the Nature Portfolio Reporting Summary linked to this article.

## Online content

Any methods, additional references, Nature Portfolio reporting summaries, source data, extended data, supplementary information, acknowledgements, peer review information; details of author contributions and competing interests; and statements of data and code availability are available at 10.1038/s41586-026-10310-3.

## Supplementary information

Supplementary Information The legends for Supplementary Tables 1–12 and Supplementary Fig. 1 (uncropped gel data).

Reporting Summary

Supplementary Tables Supplementary Tables 1–12.

## Source data

Source Data Fig. 1

Source Data Fig. 2

Source Data Fig. 3

Source Data Fig. 4

Source Data Fig. 5

Source Data Extended Data Fig. 2

Source Data Extended Data Fig. 3

Source Data Extended Data Fig. 6

Source Data Extended Data Fig. 7

Source Data Extended Data Fig. 8

Source Data Extended Data Fig. 9

Source Data Extended Data Fig. 10

## Data Availability

snRNA-seq data for E18.5 mice generated in this study are available in the GEO database under the accession number GSE314471. The snRNA-seq dataset of excitatory neurons from P26 *Cux2*^*cre*^ mice is available at Zenodo^72^ (10.5281/zenodo.18489557). snRNA-seq data of neurons from the DTA mice can be accessed at Zenodo^73^ (10.5281/zenodo.18022784). Human sequencing data generated previously^2^ are available in the Sequence Read Archive (SRA) under accession number PRJNA544731 and are viewable on the UCSF Cell Browser (https://cells.ucsc.edu/?ds=ms). Source data are provided with this paper.

## Code availability

Source code is available at GitHub (https://github.com/RowitchLab/Code_for_Cux2_Atf4_paper).

## References

[1] Fu, H., Hardy, J. & Duff, K. E. Selective vulnerability in neurodegenerative diseases. *Nat. Neurosci.* **21**, 1350–1358 (2018). 30250262 10.1038/s41593-018-0221-2 PMC6360529

[2] Schirmer, L. et al. Neuronal vulnerability and multilineage diversity in multiple sclerosis. *Nature* **573**, 75–82 (2019). 31316211 10.1038/s41586-019-1404-z PMC6731122

[3] Lassmann, H., Van Horssen, J. & Mahad, D. Progressive multiple sclerosis: pathology and pathogenesis. *Nat. Rev. Neurol.* **8**, 647–656 (2012). 23007702 10.1038/nrneurol.2012.168

[4] Mahad, D. H., Trapp, B. D. & Lassmann, H. Pathological mechanisms in progressive multiple sclerosis. *Lancet Neurol.* **14**, 183–193 (2015). 25772897 10.1016/S1474-4422(14)70256-X

[5] Schumacher, B., Pothof, J., Vijg, J. & Hoeijmakers, J. H. The central role of DNA damage in the ageing process. *Nature* **592**, 695–703 (2021). 33911272 10.1038/s41586-021-03307-7 PMC9844150

[6] Zhao, Y., Simon, M., Seluanov, A. & Gorbunova, V. DNA damage and repair in age-related inflammation. *Nat. Rev. Immunol.* **23**, 75–89 (2023). 35831609 10.1038/s41577-022-00751-y PMC10106081

[7] Maynard, S., Fang, E. F., Scheibye-Knudsen, M., Croteau, D. L. & Bohr, V. A. DNA damage, DNA repair, aging, and neurodegeneration. *Cold Spring Harb. Perspect. Med.* **5**, a025130 (2015). 26385091 10.1101/cshperspect.a025130 PMC4588127

[8] Reich, D., Lucchinetti, C. F. & Calabresi, P. A. Multiple sclerosis. *N. Engl. J. Med.* **378**, 169–180 (2018). 29320652 10.1056/NEJMra1401483 PMC6942519

[9] Magliozzi, R., Howell, O. W., Calabrese, M. & Reynolds, R. Meningeal inflammation as a driver of cortical grey matter pathology and clinical progression in multiple sclerosis. *Nat. Rev. Neurol.* **19**, 461–476 (2023). 37400550 10.1038/s41582-023-00838-7

[10] Hauser, S. L. & Cree, B. A. Treatment of multiple sclerosis: a review. *Am. J. Med.* **133**, 1380–1390 (2020). 32682869 10.1016/j.amjmed.2020.05.049 PMC7704606

[11] Yang, J. H., Rempe, T., Whitmire, N., Dunn-Pirio, A. & Graves, J. S. Therapeutic advances in multiple sclerosis. *Front. Neurol.* **13**, 824926 (2022). 35720070 10.3389/fneur.2022.824926 PMC9205455

[12] Ramdzan, Z. M., Vickridge, E., Faraco, C. C. & Nepveu, A. CUT domain proteins in DNA repair and cancer. *Cancers* **13**, 2953 (2021). 34204734 10.3390/cancers13122953 PMC8231510

[13] Munschauer, M. et al. The NORAD lncRNA assembles a topoisomerase complex critical for genome stability. *Nature* **561**, 132–136 (2018). 30150775 10.1038/s41586-018-0453-z

[14] Anderson, L., Henderson, C. & Adachi, Y. Phosphorylation and rapid relocalization of 53BP1 to nuclear foci upon DNA damage. *Mol. Cell. Biol.* **21**, 1719–1729 (2001). 11238909 10.1128/MCB.21.5.1719-1729.2001 PMC86718

[15] Mah, L., El-Osta, A. & Karagiannis, T. γH2AX: a sensitive molecular marker of DNA damage and repair. *Leukemia* **24**, 679–686 (2010). 20130602 10.1038/leu.2010.6

[16] Solier, S. & Pommier, Y. The nuclear γ-H2AX apoptotic ring: implications for cancers and autoimmune diseases. *Cell. Mol. Life Sci.* **71**, 2289–2297 (2014). 24448903 10.1007/s00018-013-1555-2 PMC4032592

[17] Traka, M., Podojil, J. R., McCarthy, D. P., Miller, S. D. & Popko, B. Oligodendrocyte death results in immune-mediated CNS demyelination. *Nat. Neurosci.* **19**, 65–74 (2016). 26656646 10.1038/nn.4193 PMC4837900

[18] Traka, M. et al. A genetic mouse model of adult-onset, pervasive central nervous system demyelination with robust remyelination. *Brain* **133**, 3017–3029 (2010). 20851998 10.1093/brain/awq247 PMC4415057

[19] Mata-Garrido, J., Casafont, I., Tapia, O., Berciano, M. T. & Lafarga, M. Neuronal accumulation of unrepaired DNA in a novel specific chromatin domain: structural, molecular and transcriptional characterization. *Acta Neuropathol. Commun.* **4**, 41 (2016). 27102221 10.1186/s40478-016-0312-9 PMC4840862

[20] Duncan, G. J. et al. Remyelination protects neurons from DLK-mediated neurodegeneration. *Nat. Commun.* **15**, 9148 (2024). 39443516 10.1038/s41467-024-53429-5 PMC11500002

[21] Xia, W. et al. Expansion of outer cortical CUX2 neurons requires adaptations for DNA repair. *Nature*10.1038/s41586-026-10290-4 (2026). 10.1038/s41586-026-10290-4 PMC13190340 41922774

[22] International Multiple Sclerosis Genetics Consortium. Multiple sclerosis genomic map implicates peripheral immune cells and microglia in susceptibility. *Science* **365**, eaav7188 (2019). 31604244 10.1126/science.aav7188 PMC7241648

[23] Dueva, R. & Iliakis, G. Replication protein A: a multifunctional protein with roles in DNA replication, repair and beyond. *NAR Cancer* **2**, zcaa022 (2020). 34316690 10.1093/narcan/zcaa022 PMC8210275

[24] Gingras, H., Cases, O., Krasilnikova, M., Bérubé, G. & Nepveu, A. Biochemical characterization of the mammalian Cux2 protein. *Gene* **344**, 273–285 (2005). 15656993 10.1016/j.gene.2004.11.008

[25] Akyuva, Y., Nazıroğlu, M. & Yıldızhan, K. Selenium prevents interferon-gamma induced activation of TRPM2 channel and inhibits inflammation, mitochondrial oxidative stress, and apoptosis in microglia. *Metab. Brain Dis.* **36**, 285–298 (2021). 33044639 10.1007/s11011-020-00624-0

[26] Sheng, W. S., Hu, S., Feng, A. & Rock, R. B. Reactive oxygen species from human astrocytes induced functional impairment and oxidative damage. *Neurochem. Res.* **38**, 2148–2159 (2013). 23918204 10.1007/s11064-013-1123-z PMC3798006

[27] Spencer, N. G., Schilling, T., Miralles, F. & Eder, C. Mechanisms underlying interferon-γ-induced priming of microglial reactive oxygen species production. *PLoS ONE* **11**, e0162497 (2016). 27598576 10.1371/journal.pone.0162497 PMC5012572

[28] Monteiro, S., Roque, S., Marques, F., Correia-Neves, M. & Cerqueira, J. J. Brain interference: revisiting the role of IFNγ in the central nervous system. *Prog. Neurobiol.* **156**, 149–163 (2017). 28528956 10.1016/j.pneurobio.2017.05.003

[29] Polyzos, A. A. et al. Base excision repair and double strand break repair cooperate to modulate the formation of unrepaired double strand breaks in mouse brain. *Nat. Commun.* **15**, 7726 (2024). 39231940 10.1038/s41467-024-51906-5 PMC11375129

[30] Zoupi, L. et al. Selective vulnerability of inhibitory networks in multiple sclerosis. *Acta Neuropathol.* **141**, 415–429 (2021). 33449171 10.1007/s00401-020-02258-z PMC7882577

[31] Anusha-Kiran, Y. et al. Regional heterogeneity in mitochondrial function underlies region specific vulnerability in human brain ageing: Implications for neurodegeneration. *Free Radic. Biol. Med.* **193**, 34–57 (2022). 36195160 10.1016/j.freeradbiomed.2022.09.027

[32] Cabré, R. et al. Specific metabolomics adaptations define a differential regional vulnerability in the adult human cerebral cortex. *Front. Mol. Neurosci.* **9**, 138 (2016). 28008307 10.3389/fnmol.2016.00138 PMC5143679

[33] Fischer, M. T. et al. Disease-specific molecular events in cortical multiple sclerosis lesions. *Brain* **136**, 1799–1815 (2013). 23687122 10.1093/brain/awt110 PMC3673462

[34] Lu, F. et al. Oxidative damage to mitochondrial DNA and activity of mitochondrial enzymes in chronic active lesions of multiple sclerosis. *J. Neurol. Sci.* **177**, 95–103 (2000). 10980305 10.1016/s0022-510x(00)00343-9

[35] Vladimirova, O. et al. Oxidative damage to DNA in plaques of MS brains. *Mult. Scler.* **4**, 413–418 (1998). 9839301 10.1177/135245859800400503

[36] Motyer, A. et al. Neuronal somatic mutations are increased in multiple sclerosis lesions. *Nat. Neurosci.*10.1038/s41593-025-01895-5 (2025). 10.1038/s41593-025-01895-5 40038527

[37] Yang, J.-H. et al. Loss of epigenetic information as a cause of mammalian aging. *Cell* **186**, 305–326 (2023). 36638792 10.1016/j.cell.2022.12.027 PMC10166133

[38] Pal, R. et al. CUX2 protein functions as an accessory factor in the repair of oxidative DNA damage. *J. Biol. Chem.* **290**, 22520–22531 (2015). 26221032 10.1074/jbc.M115.651042 PMC4566227

[39] Komulainen, E. et al. Parp1 hyperactivity couples DNA breaks to aberrant neuronal calcium signalling and lethal seizures. *EMBO Rep.* **22**, e51851 (2021). 33932076 10.15252/embr.202051851 PMC8097344

[40] Barington, M., Risom, L., Ek, J., Uldall, P. & Ostergaard, E. A recurrent de novo CUX2 missense variant associated with intellectual disability, seizures, and autism spectrum disorder. *Eur. J. Hum. Genet.* **26**, 1388–1391 (2018). 29795476 10.1038/s41431-018-0184-5 PMC6117349

[41] Suzuki, T. et al. CUX2 deficiency causes facilitation of excitatory synaptic transmission onto hippocampus and increased seizure susceptibility to kainate. *Sci. Rep.* **12**, 6505 (2022). 35581205 10.1038/s41598-022-10715-w PMC9114133

[42] Mukherjee, C. et al. Oligodendrocytes provide antioxidant defense function for neurons by secreting ferritin heavy chain. *Cell Metab.* **32**, 259–272 (2020). 32531201 10.1016/j.cmet.2020.05.019 PMC7116799

[43] Calabrese, M. et al. Exploring the origins of grey matter damage in multiple sclerosis. *Nat. Rev. Neurosci.* **16**, 147–158 (2015). 25697158 10.1038/nrn3900

[44] Trapp, B. D. & Nave, K.-A. Multiple sclerosis: an immune or neurodegenerative disorder? *Annu. Rev. Neurosci.* **31**, 247–269 (2008). 18558855 10.1146/annurev.neuro.30.051606.094313

[45] James, R. E. et al. Persistent elevation of intrathecal pro-inflammatory cytokines leads to multiple sclerosis-like cortical demyelination and neurodegeneration. *Acta Neuropathol. Commun.* **8**, 66 (2020). 32398070 10.1186/s40478-020-00938-1 PMC7218553

[46] van Olst, L. et al. Meningeal inflammation in multiple sclerosis induces phenotypic changes in cortical microglia that differentially associate with neurodegeneration. *Acta Neuropathol.* **141**, 881–899 (2021). 33779783 10.1007/s00401-021-02293-4 PMC8113309

[47] Chen, Y., Kunjamma, R. B., Weiner, M., Chan, J. R. & Popko, B. Prolonging the integrated stress response enhances CNS remyelination in an inflammatory environment. *eLife* **10**, e65469 (2021). 33752802 10.7554/eLife.65469 PMC7987340

[48] Spain, R. et al. Lipoic acid in secondary progressive MS: a randomized controlled pilot trial. *Neurol. Neuroimmunol. Neuroinflamm.* **4**, e374 (2017). 28680916 10.1212/NXI.0000000000000374 PMC5489387

[49] Monti, D. A. et al. *N*-acetyl cysteine administration is associated with increased cerebral glucose metabolism in patients with multiple sclerosis: an exploratory study. *Front. Neurol.* **11**, 88 (2020). 32117038 10.3389/fneur.2020.00088 PMC7033492

[50] Engelenburg, H. J. et al. Multiple sclerosis severity variant in DYSF-ZNF638 locus associates with neuronal loss and inflammation. *iScience* **28**, 112430 (2025). 10.1016/j.isci.2025.112430 PMC12063138 40352730

[51] International Multiple Sclerosis Genetics Consortium & Multiple MS Consortium. Locus for severity implicates CNS resilience in progression of multiple sclerosis. *Nature* **619**, 323–331 (2023). 37380766 10.1038/s41586-023-06250-x PMC10602210

[52] Ivanova, A. et al. In vivo genetic ablation by Cre-mediated expression of diphtheria toxin fragment A. *Genesis* **43**, 129–135 (2005). 16267821 10.1002/gene.20162 PMC2233880

[53] Doerflinger, N. H., Macklin, W. B. & Popko, B. Inducible site-specific recombination in myelinating cells. *Genesis* **35**, 63–72 (2003). 12481300 10.1002/gene.10154

[54] Lin, W. et al. Interferon-γ inhibits central nervous system remyelination through a process modulated by endoplasmic reticulum stress. *Brain* **129**, 1306–1318 (2006). 16504972 10.1093/brain/awl044

[55] Lin, W. et al. Interferon-γ induced medulloblastoma in the developing cerebellum. *J. Neurosci.* **24**, 10074–10083 (2004). 15537876 10.1523/JNEUROSCI.2604-04.2004 PMC6730177

[56] Franco, S. J., Martinez-Garay, I., Gil-Sanz, C., Harkins-Perry, S. R. & Müller, U. Reelin regulates cadherin function via Dab1/Rap1 to control neuronal migration and lamination in the neocortex. *Neuron* **69**, 482–497 (2011). 21315259 10.1016/j.neuron.2011.01.003 PMC3056352

[57] Gil-Sanz, C. et al. Lineage tracing using Cux2-cre and Cux2-CreERT2 mice. *Neuron* **86**, 1091–1099 (2015). 25996136 10.1016/j.neuron.2015.04.019 PMC4455040

[58] Ebert, S. M. et al. Stress-induced skeletal muscle Gadd45a expression reprograms myonuclei and causes muscle atrophy. *J. Biol. Chem.* **287**, 27290–27301 (2012). 22692209 10.1074/jbc.M112.374777 PMC3431665

[59] Li, X. et al. Deep learning enables accurate clustering with batch effect removal in single-cell RNA-seq analysis. *Nat. Commun.* **11**, 2338 (2020). 32393754 10.1038/s41467-020-15851-3 PMC7214470

[60] Wolf, F. A., Angerer, P. & Theis, F. J. SCANPY: large-scale single-cell gene expression data analysis. *Genome Biol.* **19**, 15 (2018). 29409532 10.1186/s13059-017-1382-0 PMC5802054

[61] Yao, Z. et al. A taxonomy of transcriptomic cell types across the isocortex and hippocampal formation. *Cell* **184**, 3222–3241 (2021). 34004146 10.1016/j.cell.2021.04.021 PMC8195859

[62] Zeisel, A. et al. Molecular architecture of the mouse nervous system. *Cell* **174**, 999–1014 (2018). 30096314 10.1016/j.cell.2018.06.021 PMC6086934

[63] Akhmedov, M., Martinelli, A., Geiger, R. & Kwee, I. Omics Playground: a comprehensive self-service platform for visualization, analytics and exploration of big omics data. *NAR Genomics Bioinform.* **2**, lqz019 (2020). 10.1093/nargab/lqz019 PMC7671354 33575569

[64] Raudvere, U. et al. g:Profiler: a web server for functional enrichment analysis and conversions of gene lists (2019 update). *Nucleic Acids Res.* **47**, W191–W198 (2019). 31066453 10.1093/nar/gkz369 PMC6602461

[65] Yu, G., Wang, L.-G., Han, Y. & He, Q.-Y. clusterProfiler: an R package for comparing biological themes among gene clusters. *Omics* **16**, 284–287 (2012). 22455463 10.1089/omi.2011.0118 PMC3339379

[66] Friedberg, E. C., Walker, G. C., Siede, W. & Wood, R. D. *DNA Repair and Mutagenesis* (American Society for Microbiology Press, 2005).

[67] Lange, S. S., Takata, K. -i & Wood, R. D. DNA polymerases and cancer. *Nat. Rev. Cancer* **11**, 96–110 (2011). 21258395 10.1038/nrc2998 PMC3739438

[68] Wood, R. D., Mitchell, M., Sgouros, J. & Lindahl, T. Human DNA repair genes. *Science* **291**, 1284–1289 (2001). 11181991 10.1126/science.1056154

[69] Yusa, K. et al. Targeted gene correction of α1-antitrypsin deficiency in induced pluripotent stem cells. *Nature* **478**, 391–394 (2011). 21993621 10.1038/nature10424 PMC3198846

[70] Rajendra, E. et al. Quantitative, titratable and high-throughput reporter assays to measure DNA double strand break repair activity in cells. *Nucleic Acids Res.* **52**, 1736–1752 (2024). 38109306 10.1093/nar/gkad1196 PMC10899754

[71] Cubelos, B. et al. Cux1 and Cux2 regulate dendritic branching, spine morphology, and synapses of the upper layer neurons of the cortex. *Neuron* **66**, 523–535 (2010). 20510857 10.1016/j.neuron.2010.04.038 PMC2894581

[72] Morcom, L. et al. Data for ‘DNA damage burden causes selective CUX2 neuron loss in neuroinflammation’. *Zenodo*10.5281/zenodo.18489557 (2026). 10.1038/s41586-026-10310-3 PMC13190333 41922773

[73] Morcom, L. et al. Data for ‘DNA damage burden causes selective CUX2 neuron loss in neuroinflammation’. *Zenodo*10.5281/zenodo.18022784 (2026). 10.1038/s41586-026-10310-3 PMC13190333 41922773

