# Supplementary material for: DNA damage burden causes selective CUX2 neuron loss in neuroinflammation
Source: Nature. 2026 Apr 1;653(8115):809–18. doi: 10.1038/s41586-026-10310-3 (PMC13190333; doi:10.1038/s41586-026-10310-3)
Supplement: Supplementary file 1 — The legends for Supplementary Tables 1–12 and Supplementary Fig. 1 (uncropped gel data). [file 41586_2026_10310_MOESM1_ESM.pdf]

nature portfolio

https://doi.org/10.1038/s41586-026-10310-3

Supplementary information

DNA damage burden causes selective CUX2 neuron loss in neuroinflammation

In the format provided by the
authors and unedited

Supplementary information Guide

DNA damage burden causes selective CUX2 neuron loss in neuroinflammation

Laura Morcom[1,2§], Wenlong Xia[3§], Zhaoyang Xu[1,4], Yashika Awasthi[5], Matthew O Ellis[2], Celine Geywitz[6], Tomas Noli[6],Amel Zulji[6], Daniel Yamamoto[1], Gemma C Girdler[1], Li Kai[5], Keying Zhu[3], Mingming Wei[3], Xiao-Yan Tang[3], Kimberly K. Hoi[3], Julio Gonzalez Maya[3], Greg J Duncan[7], Adrien M Vaquie[1], Diana Gold Diaz[1], Riki Kawaguchi[8,9], Erdong Liu[5], Yu Sun[2], Denny Yang[2], Gregory Jordan[1], I-ling Lu[10,11] , Staffan Holmqvist[1], Theresa Bartels[1], Katherine Ridley[1], Jennifer Ja-Yoon Choi[12], Santos J Franco[13], Eric J Huang[12], Ben Emery[14], Daniel Geschwind[9,15,16], Lucas Schirmer[6,17,18], Gabriel Balmus[2,19], Brian Popko[5], Stephen P.J. Fancy[3,] and David H. Rowitch[1, 4, 10, 11*]*

Supplementary figure 1: Uncropped gel data

a, Uncropped western blots for ATM, phosphorylated ATM at S1981 (p-ATM) and Beta-tubulin in *NGN2*-iNs at day 34 of differentiation, untreated (UT) or treated with 50ng/mL IFNγ for 3 hours (*n*=4). Blue boxes indicate all lanes that were used for quantification. Red boxes indicate lanes used for display within Fig. 4d. After transfer, blots were cut as shown to facilitate multiple antibody blotting on the same samples.

Supplementary table 1: Clinical findings and subject details for human MS and neurotypical control samples. Table describes clinical findings and subject details from human samples used for immunofluorescence (IF) in Fig.1b or RNAscope in Extended data Fig. 7b. F = female, M = male.

Supplementary table 2: Curated DNA damage response (DDR) genes. Gene identification information for DDR genes compiled from GO terms GO:0006281 DNA repair and HALLMARK_DNA_REPAIR (M5898), and consolidating with previously curated "Human DNA Repair Genes" by R.Wood and M. Lowery.

Supplementary table 3: Gene ontology terms for curated DNA damage response (DDR) genes. Gene ontology results derived from g.profiler analysis on DDR genes from Supplementary table 2. Statistical enrichment was calculated using the g.SCS test with experiment-wide threshold of a=0.05.

Supplementary table 4: Differentially expressed genes in L2/3 excitatory neuron cluster of Plp-creERT2 x ROSA26- Diphtheria toxin A (DTA)-STOP-floxed mice.

Differentially expressed genes in L2/3ENs across timepoints in DTA mice (*n*=5 per group 5/6W; *n*=3 per group 27/29W and 41/44W). Significantly DEGs were identified using Wilcoxin rank sum test with FDR 0.05 and log2 fold change 0.1.

Supplementary table 5: Gene ontology for biological process (GOBP) terms enriched in L2/3 EN DTA DEGs at 27/29 weeks post induction.

Gene ontology enrichment results for L2/3 EN DTA DEGs at 27/29 weeks post induction (*n*=3 per group). See also Supplementary table 4. Enrichment significance was assessed using thresholds of $p < 0.05$ and $q < 0.05$.

Supplementary table 6: Differentially expressed genes in L2/3 excitatory neuron cluster of E18 *Cux2*$^{cre/cre}$ mice versus wildtype control

Differentially expressed genes in E18 L2/3ENs from *Cux2*^{cre} mice (*n*=3 per group). Significantly DEGs were identified using Wilcoxin rank sum test with FDR 0.05 and log2 fold change 0.1.

Supplementary table 7: Differentially expressed genes in L2/3 excitatory neuron cluster of P26 *Cux2*^{cre} mice

Differentially expressed genes in P26 L2/3ENs from *Cux2*^{cre} mice (*n*=3 per group). Significantly DEGs were identified using Wilcoxin rank sum test with FDR 0.05 and log2 fold change 0.1.

Supplementary table 8: Gene ontology for biological process (GOBP) terms enriched in P26 L2/3EN *Cux2*^{cre} versus control

Gene ontology enrichment results for DEG from P26 L2/3ENs from *Cux2*^{cre} mice (*n*=3 per group). See also Supplementary table 7. Enrichment significance was assessed using thresholds of $p < 0.05$ and $q < 0.05$.

Supplementary table 9: Differentially expressed genes in L2/3 excitatory neuron cluster of E18 *Cux2^{cre};Atf4^{fl}* mice

Differentially expressed genes in E18 L2/3ENs from *Cux2^{cre};Atf4^{fl}* mice (*n*=3 per group). Significantly DEGs were identified using Wilcoxin rank sum test with FDR 0.05 and log2 fold change 0.1.

Supplementary table 10: Pathology and disease course of mouse models of L2/3 neurodegeneration

Summary of pathological findings from DTA, *Myrf* cKO, AS-IFNγ and experimental neuroinflammation or demyelination models in *Cux2^{cre} or Cux2^{creER};Atf4^{fl}* mice. P = postnatal, wk = week.

Supplementary table 11: Primary and secondary antibodies used in the study

Descriptive summary of primary and secondary antibodies used in this study, including their source and working dilution.

Supplementary table 12: Recombinant DNA and oligonucleotides used in the study

Descriptive summary of recombinant DNA and oligonucleotides used in this study and their source.

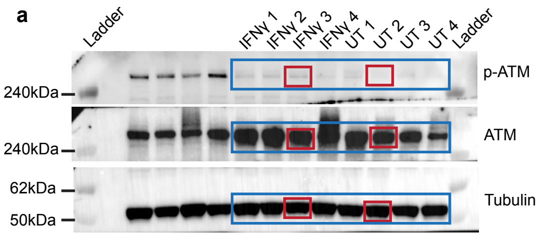

Supplementary figure 1: Uncropped gel data

a, Uncropped western blots for ATM, phosphorylated ATM at S1981 (p-ATM) and Beta-tubulin in NGN2-iNs at day 34 of differentiation, untreated (UT) or treated with 50ng/mL IFNγ for 3 hours (n=4). Blue boxes indicate all lanes that were used for quantification. Red boxes indicate lanes used for display within Fig. 4d. After transfer, blots were cut as shown to facilitate multiple antibody blotting on the same samples.
