# Supplementary material for: DNA damage burden causes selective CUX2 neuron loss in neuroinflammation
Source: Nature. 2026 Apr 1;653(8115):809–18. doi: 10.1038/s41586-026-10310-3 (PMC13190333; doi:10.1038/s41586-026-10310-3)
Supplement: Supplementary file 2 — Reporting Summary [file 41586_2026_10310_MOESM2_ESM.pdf]

nature portfolio

Corresponding author(s): David Rowitch
Stephen Fancy

Last updated by author(s): 06/02/2026

Reporting Summary

Nature Portfolio wishes to improve the reproducibility of the work that we publish. This form provides structure for consistency and transparency in reporting. For further information on Nature Portfolio policies, see our Editorial Policies and the Editorial Policy Checklist.

Statistics

For all statistical analyses, confirm that the following items are present in the figure legend, table legend, main text, or Methods section.

n/a	Confirmed	
☐	☒	The exact sample size (*n*) for each experimental group/condition, given as a discrete number and unit of measurement
☐	☒	A statement on whether measurements were taken from distinct samples or whether the same sample was measured repeatedly
☐	☒	The statistical test(s) used AND whether they are one- or two-sided<br>*Only common tests should be described solely by name; describe more complex techniques in the Methods section.*
☐	☒	A description of all covariates tested
☐	☒	A description of any assumptions or corrections, such as tests of normality and adjustment for multiple comparisons
☐	☒	A full description of the statistical parameters including central tendency (e.g. means) or other basic estimates (e.g. regression coefficient) AND variation (e.g. standard deviation) or associated estimates of uncertainty (e.g. confidence intervals)
☐	☒	For null hypothesis testing, the test statistic (e.g. *F*, *t*, *r*) with confidence intervals, effect sizes, degrees of freedom and *P* value noted<br>*Give P values as exact values whenever suitable.*
☒	☐	For Bayesian analysis, information on the choice of priors and Markov chain Monte Carlo settings
☒	☐	For hierarchical and complex designs, identification of the appropriate level for tests and full reporting of outcomes
☒	☐	Estimates of effect sizes (e.g. Cohen's *d*, Pearson's *r*), indicating how they were calculated

Our web collection on statistics for biologists contains articles on many of the points above.

Software and code

Policy information about availability of computer code

Data collection	Zen Blue Pro (v2.6), Harmony (v4.9), EVOS FL upright microscope (built in hardware) and Leica Application Suite X (v3.5.7.23225) were used for image acquisition. Acapella (v5.3.1) was used for stitching Harmony images.<br>ImageLab v.6.1 was used to collect Western blot images. A Promega 96 Microplate Glomax Luminometer and Spectrostar Nano (firmware v1.11 and software v2.12) were used to collect luminescence and absorbance data. Quantitative comparative PCR was acquired on Quantstudio 12K Flex Real-time PCR system.
Data analysis	Software and algorithms used in the study as described in the Methods: CellRanger v7.0.1 and v8.0.1 embryonic and postnatal datasets respectively, Deep Embedding for Single-cell clustering (DESC) v2.1.1 package, scanpy v1.8.1, Pegasus v1.8.1, Omics playground v2.8.19; Hiplot (ORG) Volcano plot APP v0.1.0, ClusterProfiler v4.14.6, Cellxgene v3.0, Prism v10.4.1, g.profiler v4.2.8, Fiji v2.14.0/1.54f , ggplot2 v3.5.1, FlowJo v10.7.1, CellProfiler v4.2.8, Adobe Photoshop v26.4.0 and Illustrator v30.1. Source code is available on Github: (https://github.com/RowitchLab/Code_for_Cux2_Atf4_paper).

For manuscripts utilizing custom algorithms or software that are central to the research but not yet described in published literature, software must be made available to editors and reviewers. We strongly encourage code deposition in a community repository (e.g. GitHub). See the Nature Portfolio guidelines for submitting code & software for further information.

Data

Policy information about availability of data

All manuscripts must include a data availability statement. This statement should provide the following information, where applicable:

- Accession codes, unique identifiers, or web links for publicly available datasets
- A description of any restrictions on data availability
- For clinical datasets or third party data, please ensure that the statement adheres to our policy

snRNA-seq for E18.5 mice generated in this study is available in the GEO database under the accession number GSE314471. snRNA-seq dataset of excitatory neurons from P26 Cux2cre mice is available at Zenodo: 10.5281/zenodo.18489557. snRNA-seq data of neurons from the DTA mice can be accessed at Zenodo: 10.5281/zenodo.18022784. Human sequencing data generated previously (Schirmer et al., 2019) is available in the Sequence read archive (SRA) under accession number PRJNA544731 and viewable on the UCSF Cell Browser (https://cells.ucsc.edu/?ds=ms).

Research involving human participants, their data, or biological material

Policy information about studies with human participants or human data. See also policy information about sex, gender (identity/presentation), and sexual orientation and race, ethnicity and racism.

Reporting on sex and gender	Sex/Gender was not considered due to the low sample size of the available samples but is reported in Supplementary table 1.
Reporting on race, ethnicity, or other socially relevant groupings	N/A
Population characteristics	Chronic multiple sclerosis patient samples were acquired from the UK Multiple Sclerosis Tissue Bank at Imperial College London and were 2 males and 2 females, aged 45, 50, 53, and 42. Neurotypical controls were 3 females and 2 males aged 63, 69, 61, 84, and 84. For the analysis of CUX2 and ATF4 expression in the human brain, a 25 year old male patient sample was sourced from University of California San Franscisco's Pediatric Neuropathology Research Laboratory.
Recruitment	N/A
Ethics oversight	Ethics approval for the use of human tissue was granted by the National Research Ethics Committee in the UK (08/MRE09/31) and the University of California San Franscisco Institutional Review Board.

Note that full information on the approval of the study protocol must also be provided in the manuscript.

Field-specific reporting

Please select the one below that is the best fit for your research. If you are not sure, read the appropriate sections before making your selection.

☒ Life sciences ☐ Behavioural & social sciences ☐ Ecological, evolutionary & environmental sciences

For a reference copy of the document with all sections, see nature.com/documents/nr-reporting-summary-flat.pdf

Life sciences study design

All studies must disclose on these points even when the disclosure is negative.

Sample size	Sample size was determined based on the likelihood of phenotypic effects observed in previous studies from the same human samples and animal models used (Schirmer et al., 2019; Ivanova et al., 2005; Doerflinger et al., 2003; Traka et al., 2010 and 2016; Duncan et al, 2024; Lin et al., 2004 and 2006; Ebert et al., 2012; Cubelos et al., 2010), while also considering the availability of sex and age matched samples. All sample sizes are individually reported in figure legends.
Data exclusions	A neurotypical human patient sample was omitted since the brain showed Alzheimer's pathology and cortical atrophy.
Replication	All data was produced from independent repeated experiments using biological replicates as stated in the figure legends and methods.
Randomization	All samples were randomized with the exception that equal male and female mice were allocated to experimental groups amongst littermates.
Blinding	Experimenters were not fully blinded to group allocation during data collection, but sample IDs without group information were used to deter bias and all experimental parameters were kept consistent across groups, so there are no differences between replicates/groups. Experimenters were blinded to the experimental parameters during data analyses unless the analyses involved automated quantification whereby experimental parameters were applied consistently across all samples. Two human neurotypical controls were analysed after the initial blinded analysis as one blinded sample was removed due to conflicting Alzheimer's pathology. All 7 remaining samples were quantified with the researcher blinded to the experimental condition. Subjective measurements were not used in this study.

Reporting for specific materials, systems and methods

We require information from authors about some types of materials, experimental systems and methods used in many studies. Here, indicate whether each material, system or method listed is relevant to your study. If you are not sure if a list item applies to your research, read the appropriate section before selecting a response.

Materials & experimental systems

n/a	Involved in the study
☐	☒ Antibodies
☐	☒ Eukaryotic cell lines
☒	☐ Palaeontology and archaeology
☐	☒ Animals and other organisms
☒	☐ Clinical data
☒	☐ Dual use research of concern
☒	☐ Plants

Methods

n/a	Involved in the study
☒	☐ ChIP-seq
☒	☐ Flow cytometry
☒	☐ MRI-based neuroimaging

Antibodies

Antibodies used

Chicken polyclonal anti-GFP , Abcam, Catalog # ab13970; RRID: AB_300798, 1 in 1000
Chicken polyclonal anti-mCherry, Abcam, Catalog # ab205402; RRID: AB_2722769, 1 in 1000
Chicken polyclonal anti-NeuN, EMD Millipore , Catalog # ABN91, RRID:AB_11205760, 1 in 1000
Donkey anti-Chicken IgY (H+L) Secondary Antibody, FITC, Thermo Fisher Scientific, Catalog # SA1-72000; RRID:AB_923386, 1 in 500
Donkey anti-Guinea Pig IgG (H+L), highly cross-adsorbed, CF™555 antibody , Sigma-Aldrich, Catalog # SAB4600297, RRID:AB_2814810, 1 in 500
Donkey anti-guinea pig IgG Alexa Fluor® 647-AffiniPure, Jackson ImmunoResearch, Catalog # 706-605-148, RRID:AB_2340476, 1 in 500
Donkey anti-Rabbit IgG (H+L) Highly Cross-Adsorbed Secondary Antibody, Alexa Fluor 488, Thermo Fisher Scientific, Catalog # A-21206, RRID:AB_2535792, 1 in 500
Goat polyclonal anti-ATF4, GeneTex, Catalog # GTX89973, RRID:AB_10725702, 1 in 500
Goat polyclonal anti-GFAP, Abcam, Catalog # ab53554, RRID:AB_880202, 1 in 1000
Goat polyclonal anti-IBA1 , Antibodies.com, Catalog # A82670; RRID:AB_2747449, 1 in 500
Guinea pig polyclonal anti-NeuN, EMD Millipore , Catalog # ABN90P, RRID:AB_2341095, 1 in 500
mouse IgM anti-A2B5 antibody , EMD Millipore , Catalog # MAB312, RRID:AB_94709, 1 in 250
Mouse monoclonal anti 8-oxo-dG, R&D systems, Catalog # 4354-MC-050, RRID:AB_1857195, 1 in 200
Mouse monoclonal anti Anti-DNA/RNA Damage antibody [15A3], Abcam, Catalog # ab62623, RRID:AB_94004, 1 in 150
Mouse monoclonal anti-ATM, phospho (Ser1981) , Millipore, Catalog # 05-740, RRID:AB_309954, 1 in 1000
Mouse monoclonal anti-Beta tubulin loading control, Thermo Fisher Scientific, Catalog # MA5-16308, RRID:AB_2537819, 1 in 1000
Mouse monoclonal anti-CALR, Millipore, Catalog # MAB1568, RRID:AB_94259, 1 in 500
Mouse monoclonal anti-GST Tag (8-326), Thermo Fisher Scientific, Catalog # MA4-004; RRID:AB_10979611, 1 in 200
Mouse monoclonal anti-phospho-Histone H2A.X (Ser139) clone JBW301, FITC conjugate, Sigma-Aldrich, Catalog # 16-202A; RRID: AB_568825, 1 in 500
Mouse monoclonal anti-PV, Millipore, Catalog # MAB1572, RRID:AB_2174013, 1 in 500
Rabbit anti-CALB, Swant, Catalog # cb38a, RRID:AB_3107026, 1 in 5000
Rabbit monoclonal anti-ATM, Abcam, Catalog # ab201022, RRID:AB_3674537, 1 in 1000
Rabbit monoclonal anti-CD3 antibody [SP162], Abcam, Catalog # ab135372, RRID:AB_2884903, 1 in 200
Rabbit polyclonal anti-53BP1 , Novus Biologicals, Catalog # NB100-304; RRID: AB_10003037, 1 in 500
Rabbit polyclonal anti-CASPR, , RRID:AB_2314220, 1 in 800
Rabbit polyclonal anti-CUX1, Proteintech, Catalog # 11733-1-AP, RRID:AB_2086995, 1 in 200
Rabbit polyclonal anti-CUX2, Bioss Antibodies , Catalog # BS-11832R RRID: AB_2217020, 1 in 1000
Rabbit polyclonal anti-DYKDDDDK Tag Antibody, Cell Signaling Tehcnology, Catalog # 2368S; RRID:AB_2217020., 1 in 200
Rabbit polyclonal anti-Neurofilament 200 , Sigma-Aldrich, Catalog # N4142; RRID:AB_477272, 1 in 500
Rat monoclonal anti-CTIP2 [25B6], Abcam, Catalog # ab18465, RRID:AB_2064130, 1 in 250
Rat monoclonal anti-MBP, Bio-Rad, Catalog # MCA409S, RRID:AB_325004, 1 in 500
Donkey anti-Chicken IgY (H+L) Highly Cross Adsorbed Secondary Antibody, Alexa Fluor™ 555, Thermo Fisher Scientific, Catalog # A78949; RRID:AB_2921071, 1 in 500
Donkey polyclonal anti-Goat IgG (H+L) Cross-Adsorbed Secondary Antibody, Alexa Fluor 488, Life Technologies , Catalog # A-11055; RRID:AB_2534102, 1 in 500
Donkey anti-Mouse IgG (H+L) Highly Cross-Adsorbed Secondary Antibody, Alexa Fluor™ 555, Thermo Fisher Scientific, Catalog # A-31570, RRID:AB_2536180, 1 in 500
Polyclonal Donkey anti-Mouse IgG (H+L) Highly Cross- Adsorbed Secondary Antibody, Alexa Fluor 488, Thermo Fisher Scientific, Catalog # A-21202; RRID:AB_141607, 1 in 500
Donkey anti-Rabbit IgG (H+L) Highly Cross-Adsorbed Secondary Antibody, Alexa Fluor™ 555, Thermo Fisher Scientific, Catalog # A-31572, RRID:AB_162543 , 1 in 500
Donkey polyclonal anti-Mouse IgG (H+L) Highly Cross- Adsorbed Secondary Antibody, Alexa Fluor 647, Thermo Fisher Scientific, Catalog # A-31571 RRID:AB_162542, 1 in 500
Donkey polyclonal anti-Rabbit IgG (H+L) Highly Cross-Adsorbed Secondary Antibody, Alexa Fluor 647, Thermo Fisher Scientific, Catalog # A-31573; RRID:AB_2536183, 1 in 500
Goat polyclonal anti-Chicken IgY (H+L) Secondary Antibody, Alexa Fluor 488, Thermo Fisher Scientific, Catalog # A-11039; RRID:

AB_2534096, 1 in 500
Goat anti-Mouse IGG (H+L) Secondary Antibody, Alexa Fluor 555, Thermo Fisher Scientific, Catalog # A-21422; RRID: AB_141822, 1 in 500
Goat anti-Rabbit IgG (H+L) Highly Cross-Absorbed Secondary Antibody, Alexa Fluor 647, Thermo Fisher Scientific, Catalog # A-21245; RRID: AB_2535813, 1 in 500
Goat Anti-Mouse IgG H&L (HRP), Abcam, Catalog # ab97023, RRID:AB_10679675, 1 in 5000
Goat Anti-Rabbit IgG H&L (HRP), Abcam, Catalog # ab6721, RRID:AB_955447, 1 in 5000

Validation

All antibodies are commercially available and information regarding validation can be found on the website for each vendor: https://www.abcam.com/en-us/products/primary-antibodies/cux1cux2-antibody-epr26509-154-ab309139
https://www.biossusa.com/products/bs-11832r?srsltid=AfmBOoqtJiiVt6HzxObaAW6nUtTZtWojRStf0m2Ti3CsHQr_qsaWKC75
https://www.ptglab.com/products/CUX1-Antibody-11733-1-AP.htm?srsltid=AfmBOoqIf1nYnKBJ4Rmq2va3l_f_av0ICtbELC9fOW2EQ_wpsAhXOTmu
https://www.sigmaaldrich.com/GB/en/product/mm/abn91
https://www.sigmaaldrich.com/GB/en/product/mm/abn90p
https://www.abcam.com/en-us/products/primary-antibodies/ctip2-antibody-25b6-ab18465
https://www.antibodies.com/catalog/primary-antibodies/iba1-antibody-a82670
https://www.abcam.com/en-us/products/primary-antibodies/cd3-antibody-sp162-ab135372
https://www.abcam.com/en-us/products/primary-antibodies/gfap-antibody-astrocyte-marker-ab53554
https://www.novusbio.com/products/53bp1-antibody_nb100-304?srsltid=AfmBOorMVJoo7_shKTeov2gOm-15KxZCKgUMhUloeqkanEsaRCwvVwTg
https://www.sigmaaldrich.com/GB/en/product/mm/16202a?srsltid=AfmBOooxogAq6L5tYJxkAHWCEGSYd0vPkMEH7umSZ-fFSRz2xUntpbt
https://www.sigmaaldrich.com/GB/en/product/sigma/n4142
https://www.genetex.com/Product/Detail/ATF4-antibody-C-term/GTX89973?srsltid=AfmBOop6JAoAVfulkYFoTjy9eefL7fADdqnUmHo35CdF8Ya7rxMVo387
https://www.cellsignal.com/products/primary-antibodies/dykddddk-tag-antibody-binds-to-same-epitope-as-sigma-aldrich-anti-flagm2-antibody/2368?srsltid=AfmBOoq3QZ-CxwO_RwHrfDUVGxAVfWV9bkDU6BK64RSgDRgEpWOfjKpA
https://www.thermofisher.com/antibody/product/GST-Tag-Antibody-clone-8-326-Monoclonal/MA4-004
https://www.abcam.com/en-us/products/primary-antibodies/mcherry-antibody-ab205402
https://www.abcam.com/en-us/products/primary-antibodies/gfp-antibody-ab13970
https://www.bio-rad-antibodies.com/monoclonal/cow-bovine-mbp-antibody-12-mca409.html?f=s%2Fn&evCntryLang=UKen&JSESSIONID_STERLING=adummyvalue
https://www.antibodyregistry.org/AB_3107026
https://www.sigmaaldrich.com/GB/en/product/mm/mab1568
https://www.sigmaaldrich.com/GB/en/product/mm/mab1572
https://www.thermofisher.com/antibody/product/beta-Tubulin-Loading-Control-Antibody-clone-BT7R-Monoclonal/MA5-16308
https://www.sigmaaldrich.com/GB/en/product/mm/05740?srsltid=AfmBOopF5agNAhlVKRyGypqhf73nSCBQpAnpJNrZxJNttQTFK74dCoo3
https://www.abcam.com/en-us/products/primary-antibodies/atm-antibody-epr20100-chip-grade-ab201022
https://www.rndsystems.com/products/8-oxo-dg-antibody-15a3_4354-mc-050?gclsrc=aw.ds&gad_source=1&gad_campaignid=20161432922&gbraid=0AAAAAD_kmX1Uo0Kdj9Le0m9lWAWpBaXlF&gclid=Cj0KCQiA6Y7KBhCkARIsAOxhqtMeL211KfGBZjgIUqe2vO9sEf-7O4gaxiuOEptqdBAVixidANbbxYcaAuSVEALw_wcB
https://www.abcam.com/en-us/products/primary-antibodies/dna-rna-damage-antibody-15a3-ab62623
https://www.jacksonimmuno.com/catalog/products/706-605-148
https://www.sigmaaldrich.com/GB/en/product/sigma/sab4600297?srsltid=AfmBOoqtbbl776JLaSh9g5eeX2oFvkDJPKPRPyvcTTgPWQq1XJ5e0yxd
https://www.thermofisher.com/antibody/product/Donkey-anti-Rabbit-IgG-H-L-Highly-Cross-Adsorbed-Secondary-Antibody-Polyclonal/A-21206
https://www.thermofisher.com/antibody/product/Donkey-anti-Chicken-IgY-H-L-Secondary-Antibody-Polyclonal/SA1-72000
https://www.thermofisher.com/antibody/product/Donkey-anti-Chicken-IgY-H-L-Highly-Cross-Adsorbed-Secondary-Antibody-Polyclonal/A78949
https://www.thermofisher.com/antibody/product/Donkey-anti-Goat-IgG-H-L-Cross-Adsorbed-Secondary-Antibody-Polyclonal/A-11055
https://www.thermofisher.com/antibody/product/Donkey-anti-Mouse-IgG-H-L-Highly-Cross-Adsorbed-Secondary-Antibody-Polyclonal/A-31570
https://www.thermofisher.com/antibody/product/Donkey-anti-Mouse-IgG-H-L-Highly-Cross-Adsorbed-Secondary-Antibody-Polyclonal/A-21202?ef_id=Cj0KCQiA6Y7KBhCkARIsAOxhqtN2EZx_er2Z1s9xWr6ioQZx5VDhohQTbeOmCXHCatMe5ECyyoxOq4EaAq1lEALw_wcB:G:s&s_kwcid=AL!3652!3!!!!x!!!23075843881!&gad_source=1&gad_campaignid=23075844130&gbraid=0AAAAADxi_GRu16IIqioEOayR_TruyH62&gclid=Cj0KCQiA6Y7KBhCkARIsAOxhqtN2EZx_er2Z1s9xWr6ioQZx5VDhohQTbeOmCXHCatMe5ECyyoxOq4EaAq1lEALw_wcB
https://www.thermofisher.com/antibody/product/Donkey-anti-Rabbit-IgG-H-L-Highly-Cross-Adsorbed-Secondary-Antibody-Polyclonal/A-31572
https://www.thermofisher.com/antibody/product/Donkey-anti-Mouse-IgG-H-L-Highly-Cross-Adsorbed-Secondary-Antibody-Polyclonal/A-31571
https://www.thermofisher.com/antibody/product/Donkey-anti-Rabbit-IgG-H-L-Highly-Cross-Adsorbed-Secondary-Antibody-Polyclonal/A-31573
https://www.thermofisher.com/antibody/product/Goat-anti-Chicken-IgY-H-L-Secondary-Antibody-Polyclonal/A-11039
https://www.thermofisher.com/antibody/product/Goat-anti-Mouse-IgG-H-L-Cross-Adsorbed-Secondary-Antibody-Polyclonal/A-21422
https://www.thermofisher.com/antibody/product/Goat-anti-Rabbit-IgG-H-L-Highly-Cross-Adsorbed-Secondary-Antibody-Polyclonal/A-21245
https://www.abcam.com/en-us/products/secondary-antibodies/goat-mouse-igg-h-l-hrp-ab97023

https://www.abcam.com/en-us/products/secondary-antibodies/goat-rabbit-igg-h-l-hrp-ab6721
https://www.sigmaaldrich.com/GB/en/product/mm/mab312

Eukaryotic cell lines

Policy information about cell lines and Sex and Gender in Research

Cell line source(s)	HEK293T cells were sourced from ATCC (CRL-3216). SH-SY5Y Neuroblastoma cells were obtained from ATCC (CRL-2266). hTERT RPE-1 were obtained from ATCC (# CRL-4000). The IPSC CAMi014-A corrected line was sourced from the University of Cambridge via Emmanouil Metzakopian.
Authentication	No cell lines were authenticated.
Mycoplasma contamination	All cell lines tested negative for routine mycoplasma testing by PCR and rapid test.
Commonly misidentified lines (See ICLAC register)	No cell lines used in this study are registered as commonly misidentified.

Animals and other research organisms

Policy information about studies involving animals; ARRIVE guidelines recommended for reporting animal research, and Sex and Gender in Research

Laboratory animals	Fresh Wistar/Han postnatal day 5 rat heads were obtained from Charles River Laboratory (United Kingdom) for primary cultures. ROSA26-eGFP-DTA mice (Ivanova et al., 2005) were bred to the PLP/CreERT transgenic mice (Doerflinger et al., 2013) to generate the PLP/CreERT;ROSA26-eGFPDTA (DTA) male and female mice, as previously described (Traka et al., 2010 and 2016) and used for experiments at 5-51 weeks of age. Myrffl/fl were crossed to Sox10creERT mice to generate Myrf cKO as previously described (Duncan et al., 2024), which were maintained on a C57BL/6N and C57BL/6J background, and male and female mice were used for experiments at 8-20 week of age. GFAP-tTA mice were crossed with TRE-IFN- mice to generate GFAP-tTA;TRE-IFN- double-transgenic mice, as described (Lin et al., 2004 and 2006), maintained on a C57BL/6J background, and used for experiments until 60 weeks of age. Cux2tm1.1(cre)Mull (Cux2cre) and Cux2tm3.1(cre/ERT2)Mull (Cux2creER) mice (Franco et al., 2011; Gil-Sanz et al., 2015) were crossed with C57BL/6-Atf4tm1.1Cmad/J (Atf4flox) mice (Ebert et al., 2012) to generate conditional knockout animals, were maintained on a C57BL/6 background, and used for experiments until postnatal week 13.
Wild animals	No wild animals were used in this study.
Reporting on sex	Animals were used unbiased to sex.
Field-collected samples	No samples were collected in the field.
Ethics oversight	All experiments were conducted with ethical approval from Northwestern University's Animal Care and Use Committee (IACUC), the Institutional Animal Care and Use Committee of OHSU, or UCSF's Institutional Animal Care and Use Committee (IACUC).

Note that full information on the approval of the study protocol must also be provided in the manuscript.

Plants

Seed stocks	*Report on the source of all seed stocks or other plant material used. If applicable, state the seed stock centre and catalogue number. If plant specimens were collected from the field, describe the collection location, date and sampling procedures.*
Novel plant genotypes	*Describe the methods by which all novel plant genotypes were produced. This includes those generated by transgenic approaches, gene editing, chemical/radiation-based mutagenesis and hybridization. For transgenic lines, describe the transformation method, the number of independent lines analyzed and the generation upon which experiments were performed. For gene-edited lines, describe the editor used, the endogenous sequence targeted for editing, the targeting guide RNA sequence (if applicable) and how the editor was applied.*
Authentication	*Describe any authentication procedures for each seed stock used or novel genotype generated. Describe any experiments used to assess the effect of a mutation and, where applicable, how potential secondary effects (e.g. second site T-DNA insertions, mosiacism, off-target gene editing) were examined.*
